# Spatiotemporal Mapping of the Oxytocin Receptor at Single-Cell Resolution in the Postnatally Developing Mouse Brain

**DOI:** 10.1007/s12264-024-01296-x

**Published:** 2024-09-15

**Authors:** Hao Li, Ying Li, Ting Wang, Shen Li, Heli Liu, Shuyi Ning, Wei Shen, Zhe Zhao, Haitao Wu

**Affiliations:** 1 Department of Neurobiology, Beijing Institute of Basic Medical Sciences, Beijing, 100850 China; 2 Key Laboratory of Neuroregeneration, Co-innovation Center of Neuroregeneration, Nantong University, Nantong, 226019 China; 3 Chinese Institute for Brain Research, Beijing, 102206 China

**Keywords:** Oxytocin receptor, RNAscope, Expression pattern, Postnatal development

## Abstract

The oxytocin receptor (OXTR) has garnered increasing attention for its role in regulating both mature behaviors and brain development. It has been established that OXTR mediates a range of effects that are region-specific or period-specific. However, the current studies of OXTR expression patterns in mice only provide limited help due to limitations in resolution. Therefore, our objective was to generate a comprehensive, high-resolution spatiotemporal expression map of *Oxtr* mRNA across the entire developing mouse brain. We applied RNAscope *in situ* hybridization to investigate the spatiotemporal expression pattern of *Oxtr* in the brains of male mice at six distinct postnatal developmental stages (P7, P14, P21, P28, P42, P56). We provide detailed descriptions of *Oxtr* expression patterns in key brain regions, including the cortex, basal forebrain, hippocampus, and amygdaloid complex, with a focus on the precise localization of *Oxtr*^+^ cells and the variance of expression between different neurons. Furthermore, we identified some neuronal populations with high *Oxtr* expression levels that have been little studied, including glutamatergic neurons in the ventral dentate gyrus, *Vgat*^+^*Oxtr*^+^ cells in the basal forebrain, and GABAergic neurons in layers 4/5 of the cortex. Our study provides a novel perspective for understanding the distribution of *Oxtr* and encourages further investigations into its functions.

**Supplementary Information:**

The online version contains supplementary material available at 10.1007/s12264-024-01296-x.

## Introduction

Oxytocin, an evolutionarily-conserved peptide hormone, is predominantly secreted from the paraventricular nucleus and the supraoptic nucleus [1]. Oxytocin has a multitude of physiological effects across various organs [2]. The latest research has uncovered its role in the interaction between sympathetic nerves and adipose tissue in the process of lipolysis [3], thereby expanding our understanding of both its source and function. In the brain, oxytocin functions as a neuromodulator, influencing diverse emotional and behavioral aspects such as social interaction, feeding behavior, and learning processes [2]. Notably, disruptions in the oxytocin signaling pathway have been implicated in a range of neuropsychiatric disorders, including autism spectrum disorder (ASD) [4, 5]. In most cases, the function of oxytocin relies on its G protein-coupled receptor (OXTR). Mice with haploinsufficiency of OXTR show significant social defects [6]. Furthermore, OXTR also plays a critical role in early brain development, contributing to key processes like the excitatory-to-inhibitory γ-aminobutyric acid (GABA) switch [7, 8] and the plasticity of neural circuits [9, 10].

The functionality of OXTR is characterized by high specificity, both spatially and temporally. Spatially, OXTR has varying, sometimes opposing, functions in different brain regions or cell types. For example, OXTR in the hippocampus contributes to social recognition [11, 12], while activating OXTR-positive neurons in the prefrontal cortex impairs social recognition [13]. Temporally, the effects of OXTR are subject to strict timing [7, 10]; a notable example is its regulation of synaptic transmission in sensory cortices, where the outcome is inversely related to developmental stages [10]. These findings suggest the existence of a regulatory network that precisely controls OXTR function, with its expression patterns playing a pivotal role. For instance, some brain regions with abundant OXTR expression may exhibit sparse oxytocinergic innervation, and these regions take up oxytocin from cerebrospinal fluid (CSF) in a nonspecific manner [14]. In these cases, the specific functions of the oxytocin system rely on the specific expression pattern of OXTR.

Several studies have applied various methods such as immunofluorescence [15], radioligand binding [16], and reporter mice [17] to comprehend the expression patterns of OXTR in mice. However, these studies have yielded some conflicting results. For instance, immunofluorescence-based studies suggest that OXTR expression peaks at P14 in the cortex [15], whereas reporter mouse studies indicate a peak at P21 [17]. In addition, each method has its limitations. Radioligand binding, while reflecting the functional availability of OXTR, has low resolution and a limited capability of cell-type identification. Immunofluorescence and reporter mice offer higher resolution but it is difficult to reflect the expression variance between different cells. Single-cell sequencing and spatiotemporal transcriptomics can also provide *Oxtr* expression levels but lack fine spatial information. Current research has focused on the function of OXTR in specific cell types from specific brain regions at defined time points during development. Existing expression patterns cannot meet the demand. To better elucidate the mechanisms underlying the actions of OXTR, a more detailed spatiotemporal expression pattern is required.

RNAscope *in situ* hybridization (ISH) is a novel technique for the *in situ* detection of RNA molecules [18]. This technique relies on the tandem hybridization of two independent “Z” probes followed by cascade amplification so that it can achieve high sensitivity, high specificity, and single-molecule resolution [18]. RNAscope ISH can overcome the shortcomings of existing research, offering high resolution and the ability to reflect variation in expression between different cells with fine spatial information. A recent study has used RNAscope ISH to map the expression patterns of *Oxtr* in the prairie vole [19]. However, given the evident variance of OXTR expression among rodent species [1], presently there is a lack of precise comprehensive data on whole-brain OXTR expression patterns in mice. In this study, we used RNAscope ISH to investigate the spatiotemporal expression pattern of *Oxtr* in male mice across six postnatal (P) developmental stages (P7, P14, P21, P28, P42, P56). We meticulously analyzed *Oxtr* expression in some key brain regions, including cortical areas, the basal forebrain (BF), the hippocampus, and the amygdaloid complex, mainly focusing on the precise localization of *Oxtr*-positive (*Oxtr*^+^) cells and the variance in expression among different neurons. In addition, we described the developmental dynamics of expression trajectories and cell-type distributions and identified some neuronal populations with high *Oxtr* expression levels that have received limited attention.

## Methods

### Animals

C57BL/6J mice were purchased from SiBeiFu Biotechnology Co., Ltd (Beijing, China) and housed in the animal breeding facility of the Beijing Institute of Basic Medical Sciences. All experimental procedures were in accordance with protocols approved by the Institutional Animal Care and Use Committee of the Beijing Institute of Basic Medical Sciences. Animals at different developmental stages were from different cages.

### Western Blot

Mouse brain tissue was lysed in RIPA lysis buffer (Thermo Fisher Scientific, cat. no. 89900) supplemented with a 1% protease inhibitor cocktail (MCE, cat. no. HY-K0010). The lysate was centrifuged at 12000 rpm and 4°C for 15 min. The supernatant was collected and quantified by a BCA protein assay kit (Thermo Fisher Scientific, cat. no. 23227). Subsequently, the protein samples underwent electrophoresis by SDS-PAGE and transfer onto PVDF membranes (Millipore, cat. no. IPFL00010). After blocking, the membranes were incubated with primary antibodies at 37°C for 2 h, followed by a 2-h incubation with a horseradish peroxidase-conjugated secondary antibody at room temperature. Membranes were visualized using enhanced chemiluminescence (Applygen Technologies, cat. no. P1000), and the positive bands were detected using the MiniChemi610 Chemiluminescence Imaging System. Primary antibodies used for western blot were OXTR (Abclonal, A19740, 1:1000) and GAPDH (Sungene Biotech, KM9002, 1:2000).

### RNAscope *in situ* Hybridization

Mice were deeply anesthetized by 1% pentobarbital sodium (6 μL/g, *i.p.*). Then, the animal was perfused with 4℃ saline, and the brain was immediately dissected out on the ice and embedded in an optimal cutting temperature (OCT) medium. The immersed brain was quickly frozen, and stored at − 80℃ until use. A cryostat was used to cut the brain into 15-μm coronal sections, which were stored at − 80℃ and stained within 3 months. We used Multiple Fluorescent Detection Reagents v2 (ACDbio, cat. no. 323110) to apply RNAscope ISH. After washing off the OCT, the sections were baked at 60℃ for 1 h followed by fixation in 10% neutral buffered formalin for 40 min. The fixed sections were dehydrated in 50%, 70%, 100%, and 100% ethanol in sequence. During the pre-treatment stage, the sections were treated with H_2_O_2_ for 10 min and protease III for 15 min. Then we followed the manufacturer’s protocols in the subsequent steps [18]. In all experiments, Probe-Mm-Oxtr-C1 (ACDbio, cat. no. 412171) and Opal 570 reagent (Akoya Biosciences, cat. No. OP-001003) were used to detect *Oxtr* in the red channel. Probe-Mm-Slc32a1-C2 (ACDbio, cat. no. 319191-C2) and Opal 520 reagent (Akoya Biosciences, cat. No. OP-001001) were used to detect *Vgat* in the green channel. Probe-Mm-Slc17a7-C2 (ACDbio, cat. no. 416631-C2) and Opal 520 reagent (Akoya Biosciences, cat. No. OP-001001) were used to detect *Vglut1* in the green channel. Probe-Mm-Slc17a6-C2 (ACDbio, cat. no. 319171-C2) and Opal 520 reagent (Akoya Biosciences, cat. No. OP-001001) were used to detect *Vglut2* in the green channel.

### Image Capture and Quantification

The images of all sections after RNAscope ISH were captured by the TissueFAXS imaging system (TissueGnostics) using a 40× objective lens. All images were collected using the same parameters defined by a negative control and a positive control. For control slices, Positive Control Probe-Mm (ACDbio, cat. no. 320881) and Negative Control Probe-Mm (ACDbio, cat. no. 320871) were used. Before quantifying *Oxtr* expression levels, brain regions were identified manually according to the Mouse Brain in Stereotaxic Coordinates, Third Edition. When quantifying by the statistic “*Oxtr* mRNA puncta/DPAI”, the number of *Oxtr* mRNA puncta and the number of DAPI-stained puncta were each counted using the IF2 APP in StrataQuest (TissueGnostics). The ratio of *Oxtr*^+^ cells was calculated using the IF2 APP in StrataQuest (TissueGnostics). The ratio of *Vgat*^+^ cells among *Oxtr*^+^ cells, the ratio of *Oxtr*^+^ cells among *Vgat*^+^ cells, the ratio of *Vglut1/2*^+^ cells among *Oxtr*^+^ cells, and the ratio of *Oxtr*^+^ cells among *Vglut1/2*^+^ cells were obtained using the IF3 APP in StrataQuest (TissueGnostics). All the statistics were applied with the same parameters. The value of each region is the average of the left and right hemispheres. For the number of *Oxtr* mRNA puncta in a single cell, ten *Vgat*^+^, *Vgat*^–^, *Vglut1/2*^+^, or *Vglut1/2*^–^ cells per mouse were randomly selected from the target region. Then the number was counted manually. All counts were performed by an experienced researcher who was not related to the study.

### Statistical Analysis

**Fig. 1:**
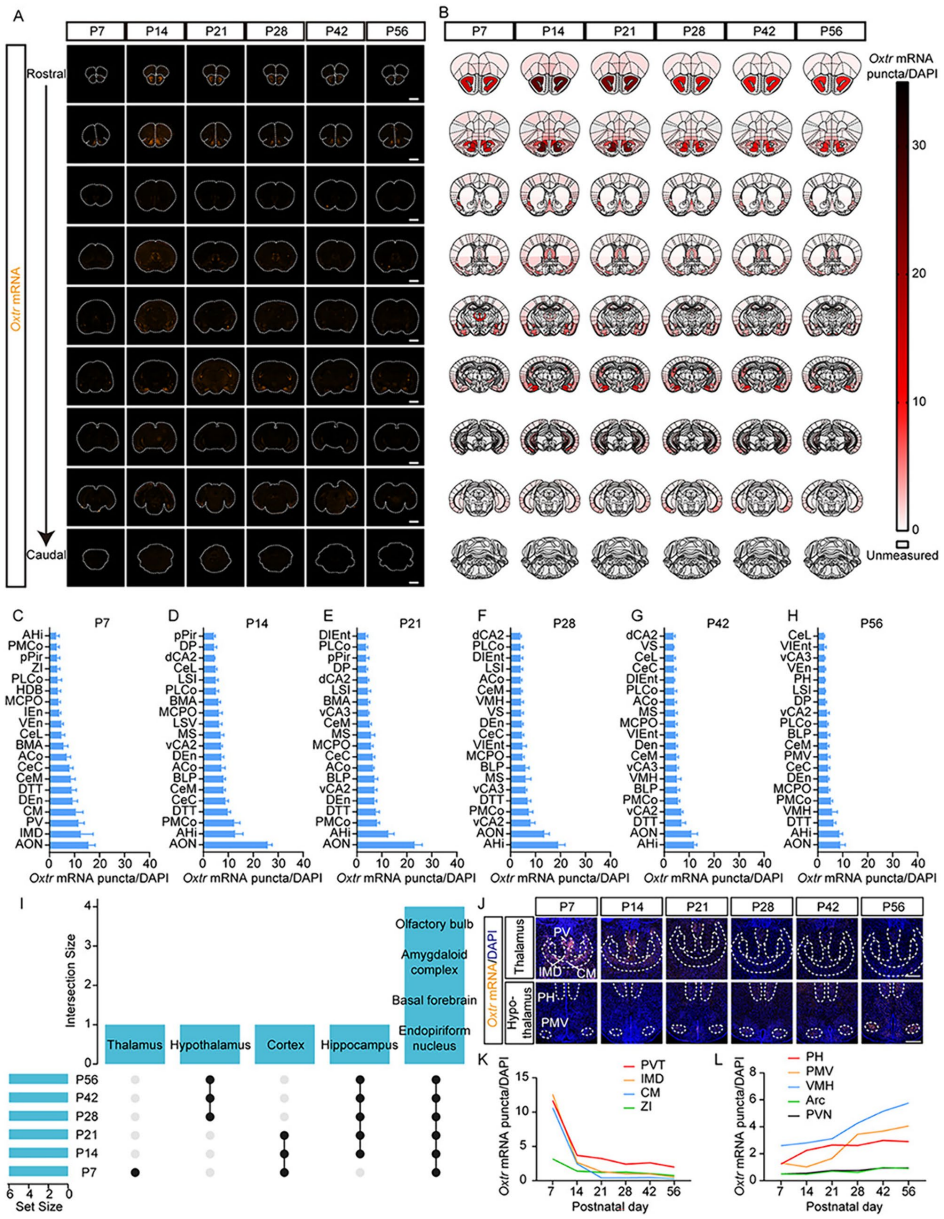
The whole-brain spatiotemporal expression pattern of *Oxtr* in mouse brain. **A** Representative coronal sections of *Oxtr* RNAscope ISH (orange) at six postnatal time points. Scale bar, 2 mm. **B** Schematics summarizing *Oxtr* expression patterns of representative sections. **C–H** The top 20 regions with the highest *Oxtr* expression levels at P7 **C**, P14 **D**, P21 **E**, P28 **F**, P42 **G**, and P56 **H** (*n =* 5). Data are presented as the mean ± SEM. **I** UpSet plot of the top 20 regions with the highest *Oxtr* expression levels at each time point. These regions were combined into large brain regions before plotting. Set size represents the number of these large brain regions with high *Oxtr* levels at each time point, and intersection size represents the number of shared regions at different time points. A black dot represents that the regions within this column highly express *Oxtr* at the time point in this row, for example, the dots in the second column indicate that *Oxtr* is highly expressed in the hypothalamus at P28, P42, and P56 (but not P7, P14, and P21) **J** Representative images of *Oxtr* RNAscope ISH (orange) from the thalamus and hypothalamus. Scale bar, 500 μm. **K**, **L** *Oxtr* expression trajectories of subregions from the thalamus **K** and hypothalamus **L** (*n =* 5). Data are presented as the mean. The full names of all abbreviations are listed in Table S1.

**Fig. 2:**
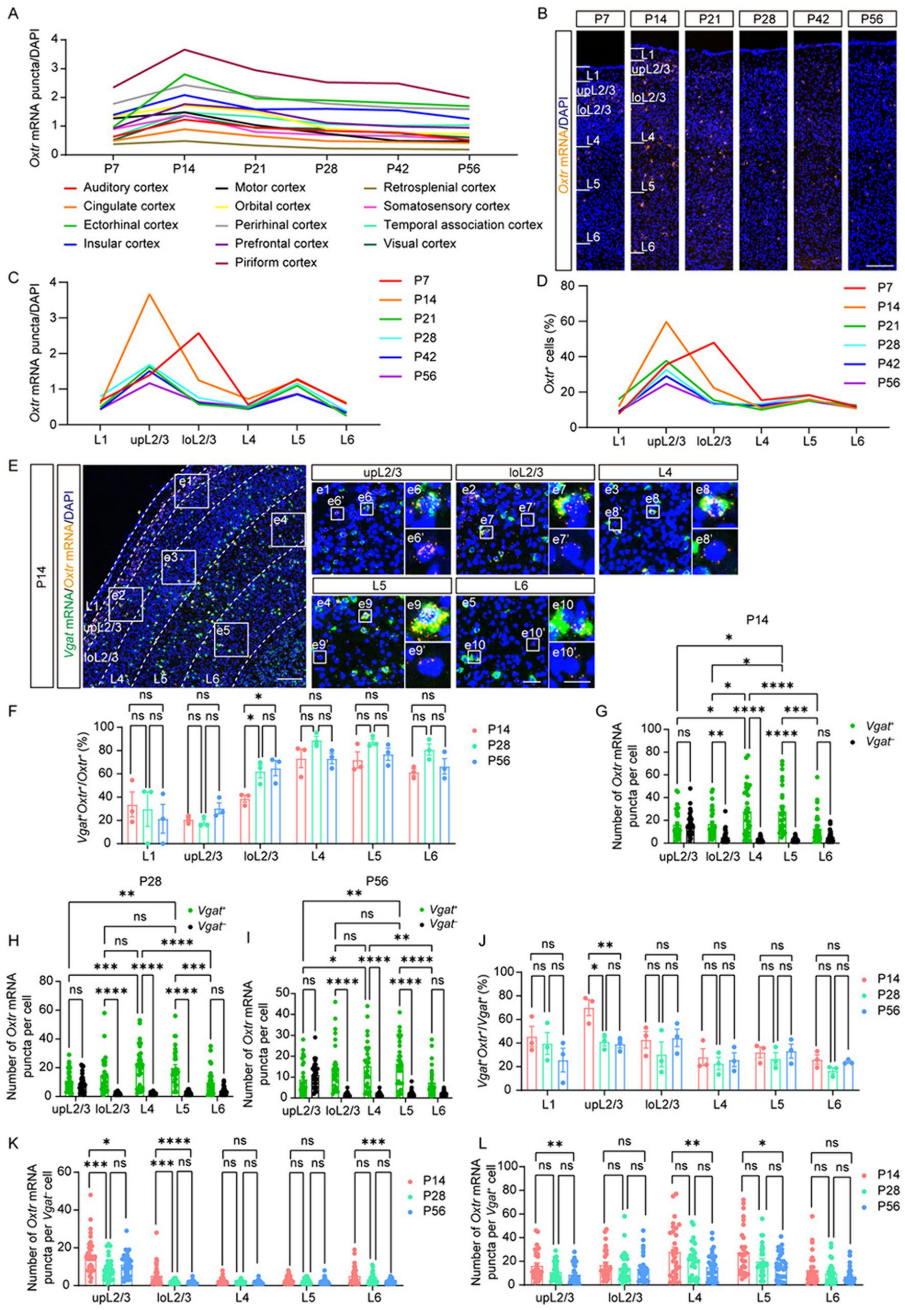
*Oxtr* expression patterns in cortices show layered characteristics. **A** *Oxtr* expression trajectories of different cortices (*n =* 5). **B** Representative images of *Oxtr* RNAscope ISH (orange) from somatosensory cortex at six periods. Scale bar, 200 μm. **C**, **D** The trajectories of “*Oxtr* mRNA puncta/DAPI” **C** or the ratio of *Oxtr*^+^ cells **D** of each layer from the somatosensory cortex at different periods (*n =* 5). **E** Representative images of *Oxtr* mRNA (orange) co-labeling with *Vgat* mRNA (green) from somatosensory cortex at P14. Scale bar, 200 μm. **e1–e5** Representative images of each layer. Scale bar, 50 μm. **e6–e10** Representative images of *Vgat*^+^ cells. **e6’–e10’** Representative images of *Vgat*^–^ cells. Scale bar, 20 μm. **F** The ratio of *Vgat*^+^ cells among *Oxtr*^+^ cells of each layer (*n =* 3). **G–I** The number of *Oxtr* mRNA puncta in different cell types and layers from the somatosensory cortex at P14 **G**, P28 **H**, and P56 **I** (*n =* 30 cells, 3 mice). **J** The ratio of *Oxtr*^+^ cells among *Vgat*^+^ cells of each layer (*n =* 3). **K**, **L** Developmental comparison of the number of *Oxtr* mRNA puncta in *Vgat*^–^ cells **K** and *Vgat*^+^ cells **L** of each layer (*n =* 30 cells, 3 mice). **A, C**, **D** Data are presented as the mean. **F–L** Data are presented as the mean ± SEM. *P <0.05, **P < 0.01, ***P < 0.001, ****P < 0.0001, one-way ANOVA with Tukey’s multiple comparisons test **F, J–L** or two-way ANOVA with Tukey’s multiple comparisons test** G–I**.

**Fig. 3:**
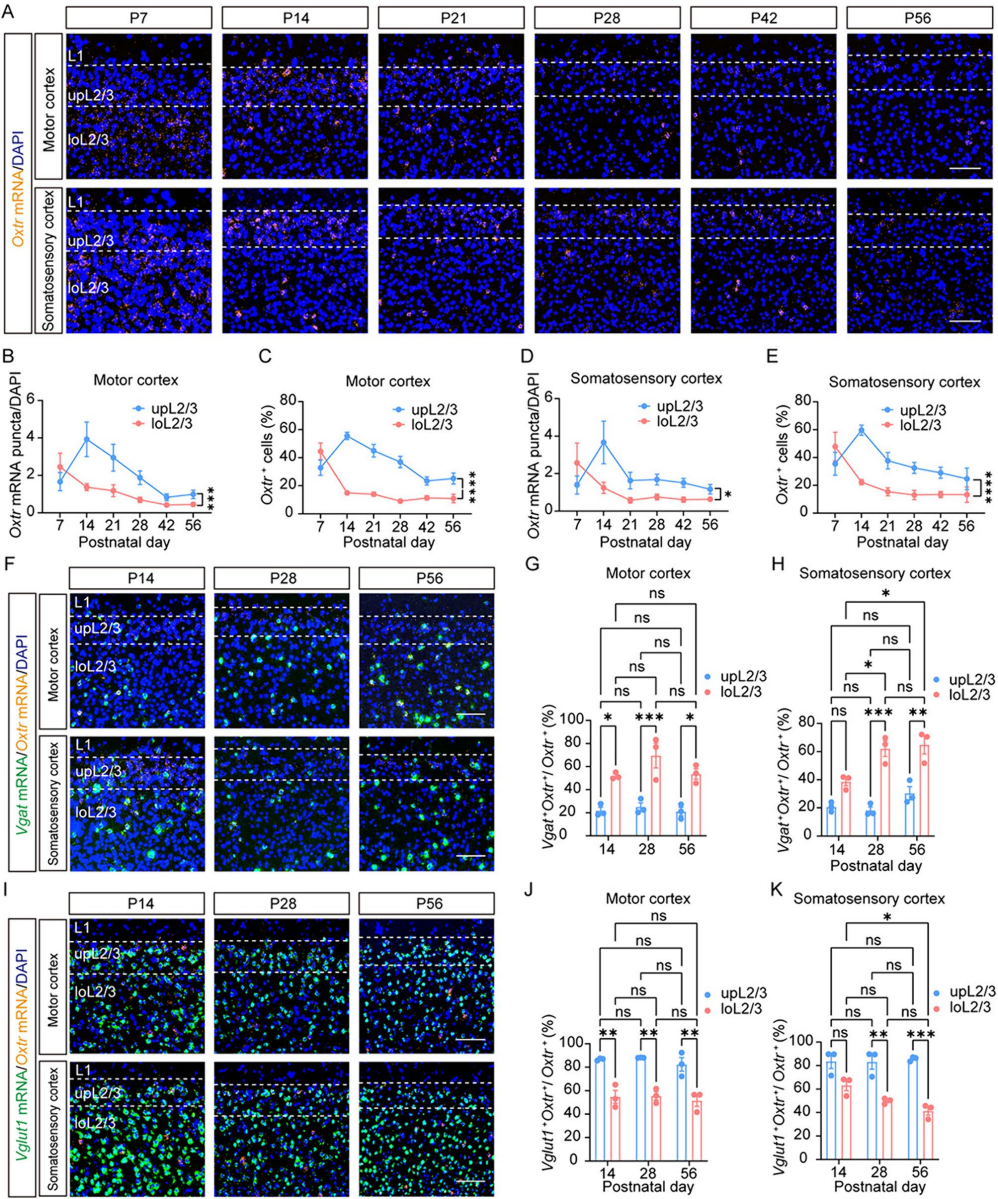
Depth-dependent variance in expression patterns of *Oxtr* within cortical L2/3. **A** Representative images of *Oxtr* mRNA (orange) from L2/3 of somatosensory cortex and motor cortex at six periods. Scale bar, 100 μm. **B–E** The trajectories of “*Oxtr* mRNA puncta/DAPI” **B** and the ratio of *Oxtr*^+^ cells **C** of L2/3 from motor cortex or somatosensory cortex **D**, **E** (*n =* 5). **F** Representative images of *Oxtr* mRNA (orange) co-labeling with *Vgat* mRNA (green) from somatosensory cortex and motor cortex at different periods. Scale bar, 100 μm. **G**, **H** The ratio of *Vgat*^+^ cells among *Oxtr*^+^ cells of L2/3 in the motor cortex **G** and somatosensory cortex **H** (*n =* 3).** I** Representative images of *Oxtr* mRNA (orange) co-labeling with *Vglut1* mRNA (green) from the somatosensory cortex and motor cortex at different periods. Scale bar, 100 μm. **J**, **K** The ratio of *Vglut1*^+^ cells among *Oxtr*^+^ cells of L2/3 in the motor cortex **J** and somatosensory cortex **K** (*n =* 3). Data are presented as the mean ± SEM. *P <0.05, **P <0.01, ***P <0.001, ****P <0.0001, two-way ANOVA with Tukey’s multiple comparisons test.

**Fig. 4:**
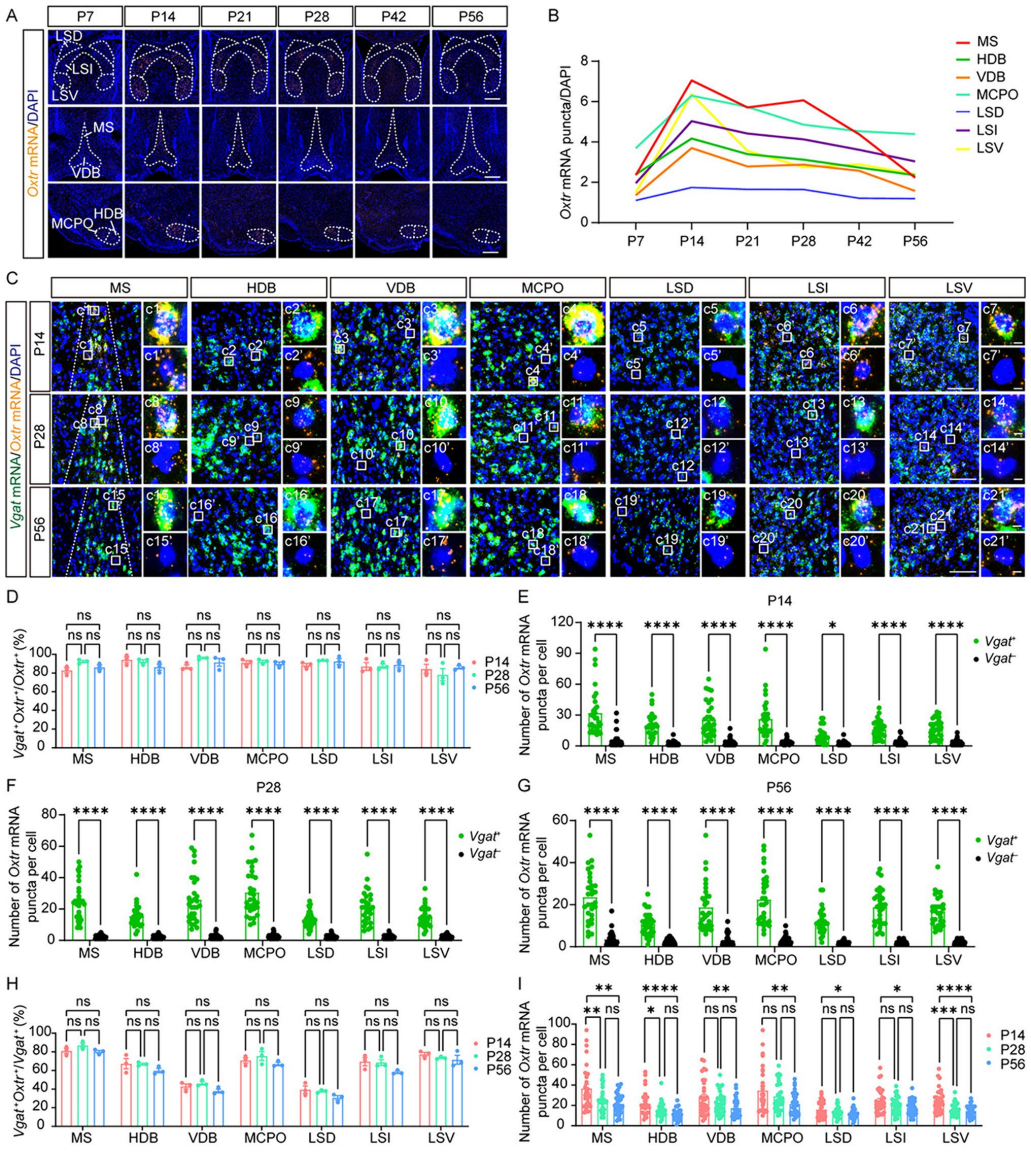
*Oxtr* is specifically expressed in GABAergic neurons in the BF. **A** Representative images of *Oxtr* mRNA (orange) from the BF at different periods. Scale bar, 500 μm. **B** The *Oxtr* expression trajectories of subregions from the BF. Data are presented as the mean. **C** Representative images of *Oxtr* mRNA (orange) co-labeling with *Vgat* mRNA (green) from the BF at different periods. Scale bar, 100 μm. **c1–c21** Representative images of *Vgat*^+^ cells. Scale bar, 5 μm. **c1’-c21’** Representative images of *Vgat*^–^ cells. Scale bar, 5 μm. **D** The ratio of *Vgat*^+^ cells among *Oxtr*^+^ cells of subregions in the BF (*n =* 3). **E-G** The number of *Oxtr* mRNA puncta in different cell types and subregions from the BF at P14 **E**, P28 **F**, and P56 **G** (*n =* 30 cells, 3 mice). **H** The ratio of *Oxtr*^+^ cells among *Vgat*^+^ cells of subregions in the BF (*n =* 3). **I** Developmental comparison of the number of *Oxtr* mRNA puncta in *Vgat*^+^ cells (*n =* 30 cells, 3 mice). **D-I** Data are presented as the mean ± SEM. *P <0.05, **P <0.01, ***P <0.001, ****P <0.0001, one-way ANOVA with Tukey’s multiple comparisons test **D, G–I** or unpaired *t-test* **E–G**. The full names of all abbreviations are listed in Table. S1.

**Fig. 5:**
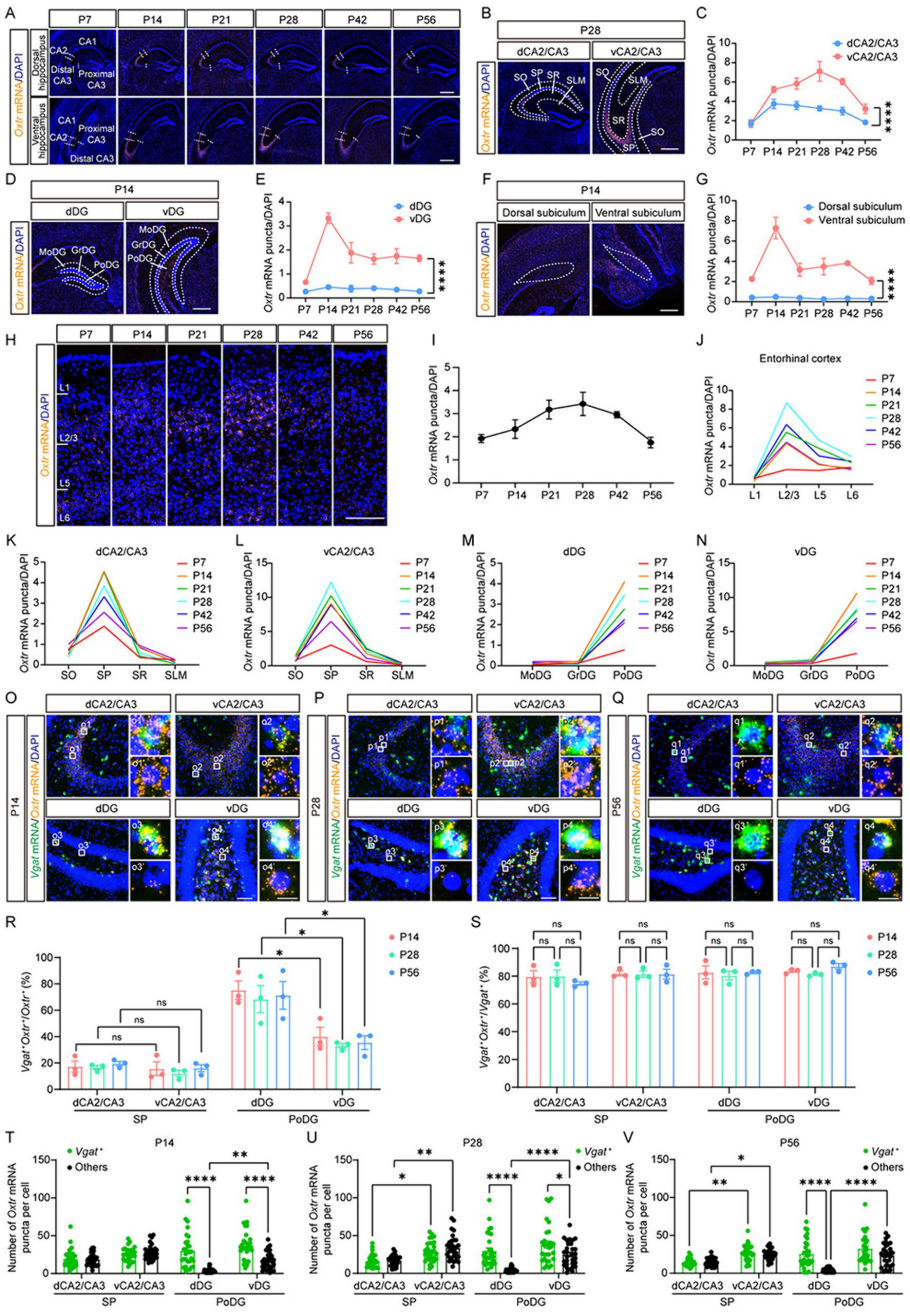
Different *Oxtr* expression patterns in the dorsal and ventral hippocampus. **A** Representative images of *Oxtr* mRNA (orange) from the hippocampus at six periods. Scale bar, 500 μm. **B–I** Representative images and quantitative expression trajectories of *Oxtr* mRNA (orange) from CA2/CA3 **(B**, **C)**, DG **(D**, **E)**, subiculum **(F**, **G)**, and entorhinal cortex **(H**, **I)** (*n =* 5). Scale bar, 500 μm **(B, D, F)** or 200 μm **(H)**. **J–N** The *Oxtr* expression trajectories of each layer from the entorhinal cortex **(J)**, dCA2/CA3 **(K)**, vCA2/CA3 **(L)**, dDG **(M)**, and vDG **(N)** (*n =* 5). Data are presented as the mean. **O–Q** Representative images of *Oxtr* mRNA (orange) co-labeling with *Vgat* mRNA (green) at P14 **(O)**, P28 **(P)**, and P56 **(Q)**. Scale bar, 100 μm. **o1–o4, p1–p4, q1–q4** Representative images of *Vgat*^+^ cells. **o1’–o4’, p1’–p4’, q1’–q4’** Representative images of *Vgat*^–^ cells. Scale bar, 20 μm. **R** The ratio of *Vgat*^+^ cells among *Oxtr*^+^ cells of different regions in the hippocampus (*n =* 3). **S** The ratio of *Oxtr*^+^ cells among *Vgat*^+^ cells of different regions in the hippocampus (*n =* 3). **T–V** The number of *Oxtr* mRNA puncta in different cell types and regions from the hippocampus at P14 **(T)**, P28 **(U)**, and P56 **(V)** (*n =* 30 cells, 3 mice). **C, E, G, R–V** Data are presented as the mean ± SEM. *P <0.05, **P <0.01, ****P <0.0001, two-way ANOVA with Tukey’s multiple comparisons test **(C, E, G, T–V)** or one-way ANOVA with Tukey’s multiple comparisons test **(S)** or unpaired *t-test* **(R)**.

**Fig. 6:**
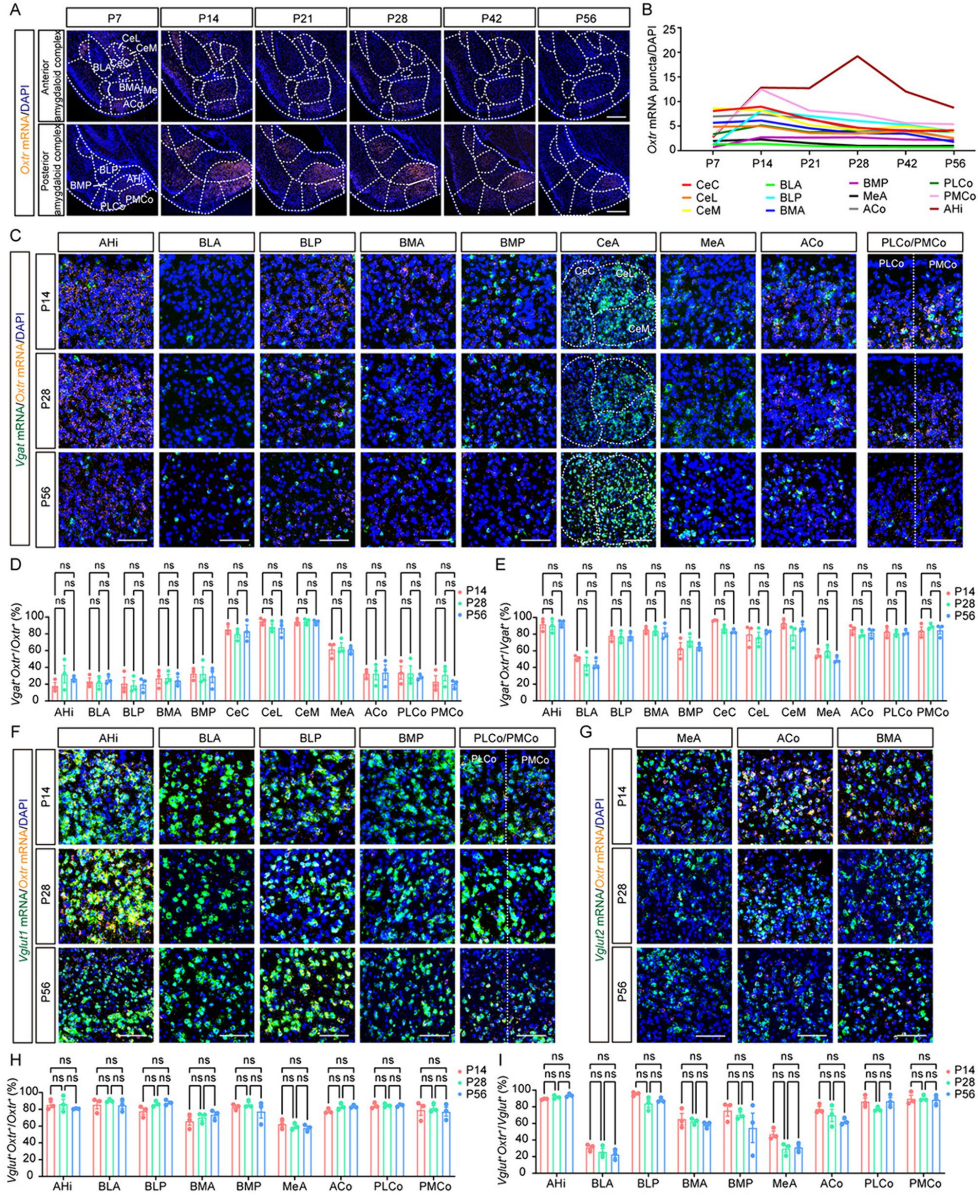
Subregion-specific distribution of *Oxtr* in the amygdaloid complex. **A** Representative images of *Oxtr* mRNA (orange) from the amygdaloid complex at six periods. Scale bar, 500 μm. **B** The *Oxtr* expression trajectories of subregions from the amygdaloid complex. Data are presented as the mean. **C** Representative images of *Oxtr* mRNA (orange) co-labeling with *Vgat* mRNA (green) at different periods. Scale bar, 100 μm. **D** The ratio of *Vgat*^+^ cells among *Oxtr*^+^ cells of subregions in the amygdaloid complex (*n =* 3). **E** The ratio of *Oxtr*^+^ cells among *Vgat*^+^ cells of subregions in the amygdaloid complex (*n =* 3).** F** Representative images of *Oxtr* mRNA (orange) co-labeling with *Vglut1* mRNA (green) at different periods. Scale bar, 100 μm. **G** Representative images of *Oxtr* mRNA (orange) co-labeling with *Vglut2* mRNA (green) at different periods (in the central amygdaloid nucleus, neither *Vglut1* nor *Vglut2* was detected, so it is not included). Scale bar, 100 μm. **H** The ratio of *Vglut*^+^ cells among *Oxtr*^+^ cells of subregions in the amygdaloid complex (*n =* 3). **I** The ratio of *Oxtr*^+^ cells among *Vglut*^+^ cells of subregions in the amygdaloid complex (*n =* 3). Data are presented as the mean ± SEM. One-way ANOVA with Tukey’s multiple comparisons test. CeA: central amygdaloid nucleus, Full names of other abbreviations are listed in Table. S1.

Blind methods were applied for experiments and statistical analyses. *Oxtr* expression schematics (Fig. 1B) were created using Prism 9 (GraphPad) with a double gradient heat map. The top 20 regions of the highest *Oxtr* expression at each developmental stage were ranked by the mean. The statistical analysis and plotting used Prism 9 (GraphPad). One-way ANOVA with Tukey’s multiple comparisons test was used to analyze developmental effects among P14, P28, and P56 (Figs 2F, 3J–L; 4D, H, I; 5S; 6E, D, H, I; S5B, F–H; S6B–E; S9B; S10D–F) and OXTR protein levels between different developmental stages (Fig. S2). An unpaired *t-test* was used to analyze the dorsal-ventral differences (Figs 5R; S10B, C) and the effect of cell type in the BF (Figs 4E–G; S9C–E). Two-way ANOVA with Tukey’s multiple comparisons was used to analyze the *Oxtr* expression patterns of specific cell types in specific layers (Figs 2G–I; S5C–E; S6F–K, cell type × layer), the developmental trend in specific layers (Fig. 3B–E, G, H, J, K, layer × time), and the difference between the dorsal and ventral hippocampus (Fig. 5C, E, G, dorsal-ventral location × time. Fig, 5T–V, dorsal-ventral location × cell type).

## Results

### The Whole-Brain Spatiotemporal Expression Pattern of *Oxtr*

To investigate the spatiotemporal pattern of whole-brain *Oxtr* expression, we selected six key postnatal developmental stages (P7, P14, P21, P28, P42, and P56). At each stage, 5 mice were sacrificed for analysis. From each mouse, a minimum of 9 brain sections were processed for RNAscope ISH (Figs 1A; S1). Subsequently, we identified regions in these sections based on stereotaxic coordinates. Consistent with previous studies, both the cerebellum and midbrain exhibited markedly low expression levels overall (Fig. 1A; Table S1) [17]. Consequently, we predominantly focused on the forebrain, selecting 111 regions for further analysis (Fig. 1B, the data of each region can be found in Table S1). For quantitative analysis, we calculated the “*Oxtr* mRNA puncta/DAPI” ratio as a metric. This statistic integrates the effects of both the ratio of *Oxtr*^+^ cells and the *Oxtr* expression level in individual *Oxtr*^+^ cells, thereby providing a more accurate reflection of expression levels in specific brain regions during distinct developmental periods. By comparing the top 20 brain regions with the highest *Oxtr* expression level at each period (Fig. 1C–H), we found some consistency within these regions.

In most periods, *Oxtr* was highly expressed in subregions of the hippocampus, olfactory bulb, BF, amygdaloid complex, and endopiriform nucleus (Figs 1I; S1). However, variations were found in some thalamic and cortical subregions, which showed elevated *Oxtr* levels in earlier developmental stages, and in parts of the hypothalamus, where *Oxtr* expression increased in later stages (Fig. 1I). Expression trajectories indicated a peak in *Oxtr* expression at P7 in the thalamus, followed by a gradual decline (Fig. 1J, K), whereas in the hypothalamus, *Oxtr* expression steadily increased (Fig. 1J, L). These findings suggest a nuanced functional role of *Oxtr* in behavioral regulation. OXTR within the ventromedial hypothalamus has recently been demonstrated to contribute to aversive social learning [20], a mature behavior for survival in a complex social group, while OXTR^+^ neurons in the paraventricular thalamus have been reported to regulate feeding motivation [21], a critical innate behavior influencing pup survival.

### The Layered Expression Patterns of *Oxtr* in Cortices

First, we specifically examined the spatiotemporal pattern of *Oxtr* expression in cortical regions, given their established role in social behaviors [13, 22–24] and cortical development [9, 10, 25]. A recent study has revealed a notably lower level of oxytocinergic innervation in comparison to *Oxtr* expression, suggesting that the predominant source of oxytocin acting on the cortex is derived from CSF [14]. This finding underscores the critical significance of the specific patterns of *Oxtr* expression in understanding the functional role of cortical *Oxtr*. Previous studies have yielded a series of data regarding the developmental trajectories of *Oxtr* expression in the cortex [15–17]. However, the results vary with methods: some studies indicated a peak at P14 [15, 16] and others at P21 [17]. Our findings corroborate the former, demonstrating that *Oxtr* expression peaks at P14 across all cortical areas (Fig. 2A). We subsequently applied Western blot analysis to corroborate our findings at the protein level. The entire cortex was dissected to facilitate detection. Our findings revealed pronounced expression during the early postnatal stage, especially at P7 and P14 (Fig. S2A, B). Furthermore, consistent results were obtained in the analysis of both the isolated somatosensory cortex and the isolated prefrontal cortex (Fig. S2C–F). In addition, we found expression heterogeneity among different cortical regions; the piriform cortex and ectorhinal cortex exhibited higher *Oxtr* levels, while lower levels were found in the retrosplenial cortex and cingulate cortex (Fig. 2A).

The typical six-layer structure of the cortex is essential for its functional role in information transmission and processing [26]. Consistent with previous studies, we found elevated *Oxtr* expression in the superficial cortical layers (Figs S3; 2B–D) [9, 16, 17]. Interestingly, in certain cortices, such as the retrosplenial and somatosensory cortex, layer (L) 5 also showed relatively higher *Oxtr* expression (Figs S3D; 2B, C), aligning with the ratio of *Oxtr*^+^ cells (Fig. 2D). Moreover, in several cortices including somatosensory, auditory, visual, and motor cortices, a depth-dependent variance was found in L2/3 (Figs S3E–G; 2B, C). Here, we conducted detailed research on the layered characteristics in the somatosensory cortex.

OXTR is expressed in various types of cells including neurons and glia. Based on the established platform for single-cell data in the Allen Brain Cell Atlas (https:// knowledge.brain-map.org/), we found that *Oxtr* is mainly expressed in neurons and its expression levels vary among different cell types (Fig. S4). This led us to investigate the distribution of *Oxtr* within distinct neuronal populations. The cortex contains two main types of neurons, namely inhibitory GABAergic neurons, and excitatory glutamatergic neurons [27]. Both types have been reported to express *Oxtr* [13, 24]. To identify the cell type of *Oxtr*^+^ cells in specific regions during development, we co-stained *Oxtr* with *Vgat*, a marker for inhibitory GABAergic neurons, or *Vglut1*, a marker for glutamatergic neurons, at P14, P28, and P56. This analysis revealed distinct cell-type distributions of *Oxtr* between layers. *Oxtr* mainly colocalized with *Vgat* in deep layers while *Vglut1* did so in superficial layers (Figs 2E, F; S5A, B). Given the distinct *Oxtr* expression levels and cell-type distributions between layers, we asked whether different types of *Oxtr*^+^ cells from different layers have different *Oxtr* expression levels. Thus, we further quantified the specific cell type from each layer in the somatosensory cortex and found variations in *Oxtr* expression levels based on cell type and layer (Fig. 2G). In upper L2/3 (upL2/3) and L6, *Vgat*-positive (*Vgat*^+^) *Oxtr*^+^ cells and *Vgat*-negative (*Vgat*^–^) *Oxtr*^+^ cells exhibited similar *Oxtr* levels (Fig. 2G). This pattern was also found between *Vglut1*-positive (*Vglut1*^+^) *Oxtr*^+^ cells and *Vglut1*-negative (*Vglut1*^–^) *Oxtr*^+^ cells (Fig. S5C), whereas in other layers, *Vgat*^+^*Oxtr*^+^ cells or *Vglut1*^–^*Oxtr*^+^ cells showed higher *Oxtr* expression (Figs 2G; S5C). Particularly, *Vgat*^+^*Oxtr*^+^ cells or *Vglut1*^–^*Oxtr*^+^ cells in L4/5 demonstrated the highest *Oxtr* expression level (Figs 2G; S5C). This layered expression pattern remained consistent across P14, P28, and P56, with the exception that *Vgat*^+^*Oxtr*^+^ cells in L4/5 expressed more *Oxtr* than *Vgat*^+^*Oxtr*^+^ cells in lower L2/3 (loL2/3) at P14 but not P28 or P56 (Fig. 2G–I). A similar phenomenon also occurred in co-staining of *Oxtr* and *Vglut1*, with *Vglut1*^–^*Oxtr*^+^ cells in L4/5 expressing more *Oxtr* than *Vglut1*^–^*Oxtr*^+^ in loL2/3 solely at P14 (Fig. S5C–E).

Given the pivotal role of the prefrontal cortex in the regulation of social behaviors, it was also meticulously analyzed. Diverging from the findings in the somatosensory cortex, *Oxtr* mainly colocalized with *Vglut1* across all layers within the prefrontal cortex (Fig. S6A–C). In addition, both GABAergic and glutamatergic neurons exhibited a comparable ratio of *Oxtr*^+^ cells, with a majority of cells in L2/3 expressing *Oxtr*, while a lower ratio of cells expressing *Oxtr* occurred in deep layers (Fig. S6D, E). However, upon quantification of *Oxtr* expression at the single-cell level, a pattern similar to the somatosensory cortex was found. Specifically, GABAergic neurons and glutamatergic neurons exhibited similar *Oxtr* levels in L2/3 and L6, while in L5 GABAergic neurons expressed more *Oxtr* than glutamatergic neurons (Fig. S6F-K). Notably, GABAergic neurons in L5 represented the neuronal population with the highest *Oxtr* level (Fig. S6F–K). Moreover, GABAergic neurons in L5 expressed more *Oxtr* than GABAergic neurons in L2/3 at P14 and P28 but not P56 (Fig. S6–K).

Having identified a commonality in the expression pattern of *Oxtr* between the somatosensory cortex and the prefrontal cortex, we then asked whether this pattern is conserved among other cortices. Extending our analysis to six additional cortices, we found that, despite variations in *Oxtr* levels across cortices (Figs S7; S8), GABAergic neurons in L5 consistently expressed more *Oxtr* than glutamatergic neurons in L5 and exhibited the highest *Oxtr* expression across all eight cortices at all three developmental time points (Figs S7; S8).

Previous studies have reported the dynamics of cortical *Oxtr* expression during development [9, 16, 17], so we analyzed the developmental changes of each layer in the somatosensory cortex and prefrontal cortex. As previously reported, we found dynamic changes in L2/3 (Fig. 2F–L) [10]. Interestingly, an increase in the ratio of *Vgat*^+^ cells among *Oxtr*^+^ cells was found exclusively in loL2/3 but not upL2/3 (Fig. 2F). Conversely, a decrease in the ratio of *Oxtr*^+^ cells among *Vgat*^+^ cells was noted only in upL2/3 but not loL2/3 (Fig. 2J). However, the ratio of *Oxtr*^+^ cells among *Vglut1*^+^ cells decreased in both upL2/3 and loL2/3 (Fig. S5F) while the ratio of *Vglut1*^+^ cells among *Oxtr*^+^ cells decreased solely in loL2/3 (Fig. S5B). Other layers maintained consistent ratios throughout development (Figs 2F, J; S5B, F). Furthermore, we noted that the *Oxtr* expression levels in *Vgat*^–^*Oxtr*^+^ cells or *Vglut1*^+^*Oxtr*^+^ cells specifically decreased in L2/3 and L6 (Figs 2K; S5G), while in *Vgat*^+^*Oxtr*^+^ cells or *Vglut1*^–^*Oxtr*^+^ cells, a specific decrease was found in upL2/3, L4, and L5. (Figs 2L; S5H). These findings suggest the multifaceted effects of *Oxtr* in specific cell types from distinct layers during development. Nevertheless, a distinct developmental pattern emerged within the prefrontal cortex, where all ratios remained consistent across all layers and stages (Fig. S6B-E). Furthermore, both GABAergic and glutamatergic neurons demonstrated a decline in *Oxtr* levels during development in L2/3 and L5, whereas, in L6, only glutamatergic neurons displayed a reduction in *Oxtr* levels throughout development (Fig. S6L, M). Our study also highlighted a depth-dependent variance of *Oxtr* expression patterns in L2/3 (Figs 2F, J; 3). We conducted detailed analyses in the motor cortex and somatosensory cortex, *Oxtr* expression levels in upL2/3 and loL2/3 followed different developmental trajectories in both cortices (Fig. 3A–E). In upL2/3, *Oxtr* expression increased from P7 to P14, followed by a gradual decrease, whereas in loL2/3, there was a continuous decrease from P7 to P56 (Fig. 3A–E). Moreover, the cell-type distributions differed in upL2/3 and loL2/3, with a higher ratio of *Vgat*^+^ cells among *Oxtr*^+^ cells in loL2/3 than in upL2/3 (Fig. 3F–H) and a higher ratio of *Vglut1*^+^ cells among *Oxtr*^+^ cells in upL2/3 than in loL2/3 (Fig. 3I–K). Our data have shown an increase in the ratio of *Vgat*^+^ cells among *Oxtr*^+^ cells and a decrease in the ratio of *Vglut1*^+^ cells among *Oxtr*^+^ cells specifically in loL2/3 (Fig. 2F). However, these dynamic changes were only found in somatosensory cortex but not motor cortex (Fig. 3F–K). These results highlight the diverse expression patterns of *Oxtr* across different cortical areas.

### *Oxtr* in the basal forebrain is predominantly expressed in *Vgat*^+^ cells

The BF is a collection of regions located in the rostroventral part of the forebrain. It plays a pivotal role in numerous neural processes, including arousal, attention, learning, and memory [28]. Previous research has identified a high level of *Oxtr* expression within the BF [2, 29]. In this study, we delineated its detailed spatiotemporal expression pattern. Throughout the six postnatal developmental periods under scrutiny, we found pronounced *Oxtr* expression in the medial septum (MS), lateral septum (LS), nucleus of the horizontal limb of the diagonal band (HDB), the nucleus of the vertical limb of the diagonal band (VDB), and the magnocellular preoptic nucleus (MCPO) (Fig. 4A). Our trajectory analysis revealed *Oxtr* expression peaked at P14 in these regions before subsequently diminishing (Fig. 4B).

Further, we endeavored to classify the cell types of *Oxtr*^+^ cells within these regions of high expression. The BF is known to comprise several different neuronal populations [30]. Our co-staining analysis indicated a predominant presence of *Vgat*^+^ cells among the *Oxtr*^+^ cells in these high-expression regions (Fig. 4C, D). Moreover, we noted that *Vgat*^+^*Oxtr*^+^ cells exhibited considerably higher levels of *Oxtr* than *Vgat*^–^*Oxtr*^+^ cells (Fig. 4C, E–G). This specific expression pattern was also validated by co-staining *Oxtr* and *Vglut2* (Fig. S9). *Oxtr* was mainly expressed in *Vglut2*^–^ cells (Fig. S9A, B) and *Vglut2*^–^*Oxtr*^+^ cells showed significantly lower levels of *Oxtr* than *Vglut2*^+^*Oxtr*^+^ cells (Fig. S9C-E). Together, our results suggested the potential of *Vgat*^+^ cells to mediate the effects of *Oxtr* in the BF.

To understand the developmental dynamics of this pattern, we examined changes over time. Both the ratio of *Vgat*^+^ cells among *Oxtr*^+^ cells and the ratio of *Oxtr*^+^ cells among *Vgat*^+^ cells remained consistent across P14, P28, and P56 (Fig. 4D, H),. However, the expression level in *Vgat*^+^*Oxtr*^+^ cells gradually decreased from P14 to P56 (Fig. 4I). These results imply a potential role for *Oxtr* in the developmental processes of the BF, highlighting the complex interplay between *Vgat*^+^ cells and oxytocin signaling in this key brain region.

### The dorsal-ventral difference of *Oxtr* expression patterns in the hippocampus

The function of OXTR in the hippocampus has been extensively studied. It is crucial for neuronal activity, network oscillatory activity, synaptic plasticity, adult neurogenesis, and social recognition memory [12]. Aberrations in OXTR expression within the hippocampus have been documented in the *Magel2* mouse model of autism [31]. Furthermore, the hippocampus has been identified as a target of oxytocin in ameliorating social behaviors in models such as bilateral whisker-trimming mice and *Fmr1*-knockout mice [32]. Given the critical role of OXTR in the hippocampus, previous studies have meticulously described its expression pattern [11, 33]. Here, we study it from a developmental perspective. Consistent with prior findings, we found high *Oxtr* expression in the CA2 and CA3 distal region [31] of both dorsal and ventral hippocampus (Fig. 5A). However, we noted a higher expression level in the ventral than in the dorsal hippocampus, with this dorsal-ventral discrepancy evident in CA2/CA3 (Fig. 5B, C), the dentate gyrus (DG) (Fig. 5D, E), and the subiculum (Fig. 5F, G). According to the expression trajectories, *Oxtr* expression in these regions generally peaked at P14, with the exception of the ventral CA2/CA3 (vCA2/CA3) where the peak occurred at P28 (Fig. 5C, E, G). Intriguingly, the expression trajectory in vCA2/CA3 is similar to that of the entorhinal cortex (Fig. 5H, I), a crucial node in the cortico-hippocampal circuit [34].

The layered structure of the hippocampus is pivotal for its function. Thus, we compared the expression levels of each layer. The entorhinal cortex predominantly expressed *Oxtr* in L2/3 (Fig. 5J). Both the dorsal and ventral parts of the hippocampus exhibited similar layer distribution patterns (Fig. 5K–N), with high *Oxtr* expression in the stratum pyramidal (SP) of CA2/CA3 (Fig. 5K, L), and the polymorphic layer of the DG (PoDG) (Fig. 5M, N). Furthermore, we identified the cell type of *Oxtr*^+^ cells. In both dorsal CA2/CA3 (dCA2/CA3) and vCA2/CA3, *Oxtr* was mainly localized in *Vgat*^–^ cells within the SP (Fig. 5O–R). This distribution pattern, however, was influenced by the low number of *Vgat*^+^ cells in the hippocampus. Our analysis revealed that most *Vgat*^+^ cells expressed *Oxtr* in both the dorsal and ventral SP (Fig. 5S), with no significant differences in *Oxtr* expression levels between *Vgat*^+^*Oxtr*^+^ cells and *Vgat*^–^*Oxtr*^+^ cells (Fig. 5T–V). At P28 and P56 (but not at P14), both *Vgat*^+^*Oxtr*^+^ cells and *Vgat*^–^*Oxtr*^+^ cells exhibited higher *Oxtr* expression in the vCA2/CA3 than in the dCA2/CA3 (Fig. 5T–V), aligning with the distinct expression trajectories (Fig. 5C).

However, a notable dorsal-ventral difference in cell-type distribution was found in the DG (Fig. 5O–R). In the dorsal DG (dDG), *Oxtr* was predominantly expressed in *Vgat*^+^ cells, while the ventral DG (vDG) showed a lower ratio of *Vgat*^+^ cells among *Oxtr*^+^ cells (Fig. 5R). Despite this, we found that most *Vgat*^+^ cells expressed *Oxtr* in both the dDG and vDG (Fig. 5S). In addition, in both the dDG and vDG, *Vgat*^+^*Oxtr*^+^ cells exhibited higher *Oxtr* expression than *Vgat*^–^*Oxtr*^+^ cells at P14 and P28 (Fig. 5T, U). Nevertheless, it is noteworthy that in vDG, *Vgat*^–^*Oxtr*^+^ cells but not *Vgat*^+^*Oxtr*^+^ cells demonstrated a higher level of *Oxtr* expression than their counterparts in the dDG (Fig. 5T–V). The expression of *Oxtr* in the DG was further validated by co-staining of *Oxtr* and *Vglut1*. *Oxtr* was mainly expressed in *Vglut1*^+^ cells in the vDG while the ratio was lower in the dDG (Fig. S10A, B), aligning with our previous results. However, differing from *Vgat*^+^ cells, the ratio of *Oxtr*^+^ cells among *Vglut1*^+^ cells was higher in the vDG than the dDG (Fig. S10C). In addition, in both the dDG and vDG, *Vglut1*^–^*Oxtr*^+^ cells showed higher *Oxtr* expression than *Vglut1*^+^*Oxtr*^+^ cells at P14 and P28 (Fig.S10D, E), while at P56 *Vglut1*^–^*Oxtr*^+^ cells and *Vglut1*^+^*Oxtr*^+^ cells had similar *Oxtr* levels in the vDG (Fig. S10F). In the vDG, *Vglut1*^+^*Oxtr*^+^ cells but not *Vglut1*^–^*Oxtr*^+^ cells demonstrated a higher level of *Oxtr* expression than their counterparts in the dDG (Fig. S10D-F).

These findings underscore the complex and region-specific expression patterns of *Oxtr* in the hippocampus, suggesting nuanced roles of *Oxtr* in different hippocampal subregions and cell types during development.

### The Subregion-Specific Distribution of *Oxtr* in the Amygdaloid Complex

Then, we focused on the amygdaloid complex, a collection of over ten nuclei situated in the temporal lobe and intricately connected to the limbic system [35]. The amygdaloid complex exhibits a diverse array of physiological functions, with each subregion exerting distinct effects [35]. Prior research has elucidated the functional heterogeneity of OXTR within this complex. For instance, OXTR in the central amygdala is involved in emotion discrimination [36], the fear response [37], and anti-anxiety [38]. OXTR in the medial amygdala is involved in sex discrimination in males [39], social recognition [40], and long-term social memory [41]. OXTR in the amygdalohippocampal area is involved in infant-directed attacks in males [42].

Our findings align with these established roles, showing high *Oxtr* expression levels in these regions (Fig. 6A). Moreover, we found high *Oxtr* expression in the basomedial amygdaloid nucleus, the cortical amygdaloid area, and the posterior part of the basolateral amygdaloid nucleus (Fig. 6A). The expression trajectories indicated that *Oxtr* expression levels peaked at P14, except in the amygdalohippocampal area, where it peaked at P28 (Fig. 6B). The amygdaloid complex is characterized by a diverse neuronal composition across its subregions [35]. In our investigation, we found that *Oxtr* in the central amygdaloid nucleus predominantly colocalized with *Vgat* (Fig. 6C, D). In the medial amygdaloid nucleus, the *Oxtr* was distributed in both GABAergic and glutamatergic neurons in an even ratio (Fig. 6C, D, G, H). *Oxtr* was mainly expressed in glutamatergic neurons in other regions of the amygdaloid complex (Fig. 6C, D, F–H). However, in all regions except the anterior part of the basolateral and medial amygdaloid nucleus, a large number of both GABAergic and glutamatergic neurons expressed *Oxtr* (Fig. 6E, I). Notably, this expression pattern was stable throughout the developmental stages examined (Fig. 6D, E, H, I).

Given that the amygdaloid complex is a sexually dimorphic region [43], we asked whether the expression pattern of *Oxtr* differs between females and males. The sexes were compared at P14, P28, and P56, and three female mice were sacrificed at each stage. We found that the variances between sexes were development-dependent. Male mice had higher *Oxtr* levels than female mice in the anterior parts of the basomedial amygdaloid nucleus and cortical amygdaloid area, the medial amygdala, and the central amygdala at P14, while at P56, solely the anterior part of the cortical amygdaloid area still exhibited a higher *Oxtr* level in males than in females (Fig. S11).

## Discussion

In this study, by applying the RNAscope ISH technique, we have elucidated the spatiotemporal expression pattern of *Oxtr* at a molecular level in the male mouse brain. By quantifying 111 regions (mainly within the forebrain) across six developmental periods, we discovered a spatial consistency in regions of high *Oxtr* expression across these intervals. We conducted a detailed analysis of the expression trajectories and the cell-type distributions of *Oxtr*^+^ cells in key areas, namely the cortical areas, the BF, hippocampus, and amygdaloid complex, where *Oxtr* demonstrates high expression and is implicated in a range of physiological effects.

Our findings indicated that in all these regions, *Oxtr* expression peaked in early postnatal developmental stages before gradually declining. First, in the cortex, the *Oxtr* expression pattern was characterized by distinct layer attributes in both developmental trajectories and cell-type distributions. Second, in the BF, *Oxtr* expression was predominantly seen in *Vgat*^+^ cells. Third, we identified notable differences between the dorsal and ventral regions of the hippocampus. Last, most importantly, we identified some relatively unexplored neuronal populations with high *Oxtr* expression levels, including glutamatergic neurons in the vDG, *Vgat*^+^*Oxtr*^+^ cells in the BF, and GABAergic neurons in cortical L5.

Oxytocin signaling mediated by OXTR is not only pivotal in the regulation of mature behaviors [13, 23, 24] but also plays a crucial role in synaptic development and neuronal activity during developmental stages [9, 10, 25]. Correspondingly, both previous studies [15] and our results showed that *Oxtr* expression peaks at P14 across multiple brain regions. This peak suggests a critical window during which oxytocin signaling *via* OXTR plays a pivotal role in shaping normal circuits. The timing coincides with a variety of significant neurodevelopmental processes like synaptic pruning and refinement [44] and experience-dependent plasticity [9] that are essential for the establishment of functional neural networks. Furthermore, the long-term impact of early-life oxytocin treatment has been reported in various models of neurodevelopmental disorders [32, 45–47]. In some models, aberrations in *Oxtr* expression were found exclusively during early postnatal periods, with no anomalies detectable in adulthood [48]. This underscores the importance of early oxytocin intervention in disorders like ASD, potentially explained by the elevated levels of *Oxtr* in early postnatal stages.

Remarkably, our research has uncovered that the *Vgat*^+^ cells in L5 remain one of the neuronal populations that exhibit the highest levels of *Oxtr* across all eight cortices at all three developmental time points. In the canonical six-layer structure of the cortex, L4 primarily functions in receiving and transmitting information to L2/3, and L5 is mainly responsible for relaying processed information from L2/3 to other cortical or subcortical areas [49, 50]. GABAergic neurons in L5 are predominantly interneurons, providing local inhibition. Previous studies have focused extensively on L2/3, but studies on interneurons in L5 are relatively limited. In the prefrontal cortex, the function of *Oxtr* in interneurons is sexually dimorphic; in males, it mediates anti-anxiety effects [51], whereas in females, it is involved in social behaviors [24]. However, the role of *Oxtr* in L5 may extend beyond the regulation of mature behaviors. Our findings suggest a developmental role for *Oxtr* in L5 interneurons, as indicated by a decline in expression from P14 to P56.

In the developing cortex, GABA is initially excitatory [52]. In addition, it can generate robust tonic GABA currents in L5 pyramidal cells [53]. Enhancements in these tonic GABA currents have been noted in the postnatal maternal separation model, a model of negative early-life events with stress reactivity and cognitive impairments [54]. Moreover, the excitatory nature of early postnatal GABA is crucial for the maturation of glutamatergic synapses *via* the activation of N-methyl-D-aspartate receptors [55]. However, by the second postnatal week, GABA transitions to inhibitory effects, with a dramatic reduction in tonic currents [53], and inhibitory GABAergic signaling interacts with mature glutamatergic signaling to maintain an excitatory-inhibitory balance [56]. The developmental switch of GABA is due to the reduction of cytoplasmic chloride, which depends on the inhibition of Na^+^–K^+^2Cl^−^ cotransporter 1 (NKCC1) and the activity of K^+^–Cl^−^ cotransporter 2 (KCC2) [56]. Oxytocin has been demonstrated to be involved in both down-regulation of NKCC1 [57] and up-regulation of KCC2 [7]. Given the critical developmental dynamics of GABAergic effects by P14 and the specific enrichment of *Oxtr* in GABAergic neurons of L5 at this stage, the developmental role of *Oxtr* in deeper cortical layers warrants further investigation.

Our study also highlights a depth-dependent variance of *Oxtr* expression patterns in L2/3, which may suggest a distinction between L2 and L3, despite previous research often treating L2/3 as a single entity due to the unclear cytoarchitectonic division in rodents [26]. Distinctions between L2 and L3 have been primarily studied in the primary auditory cortex [26, 49] and primary somatosensory barrel cortex [58–61], with noted differences in location [58] morphology [60], connective pattern [26, 61], innervation [59], and electrophysiological properties [26, 49]. We found that *Oxtr* expression patterns vary between upL2/3 and loL2/3, both in developmental trajectories and cell-type distributions, suggesting the need for further research to determine whether the depth-dependent variance represents a difference between L2 and L3.

In the BF, *Oxtr* expression is not limited to the LS, a well-known region involved in social behavior [62, 63]. High expression levels were also detected in the MS, HDB, VDB, and MCPO, regions traditionally implicated in arousal, attention, learning, and memory [28]. Oxytocin is considered an attention modulator to regulate the salience of social clues [64]. Given BF's central role in attention and motivation, *Oxtr* in these regions may contribute to the transmission of social information. In addition, while previous studies have predominantly focused on cholinergic projection neurons in the BF, it also comprises glutamatergic and GABAergic neurons. A recent study has revealed the contribution of SST^+^ GABAergic neurons to prosocial behaviors in the BF [28]. Correspondingly, our data indicates predominant *Oxtr* expression in *Vgat*^+^ cells within the BF. Moreover, aberrations in OXTR binding in the BF have been documented in the brain tissue of individuals with ASD [65], suggesting that this atypical OXTR binding may underlie abnormal social behaviors. Together, the relationship between oxytocin signaling in the BF and social behaviors merits further exploration.

The BF is known to comprise at least three neuronal populations: cholinergic, glutamatergic, and GABAergic neurons [30]. Although VGAT is usually regarded as a marker of GABAergic neurons, cholinergic neurons in the BF have also been reported to express VGAT [66] and some of their subsets have been shown to co-release GABA [67]. This mechanism of co-transmission of different neurotransmitters is important for the regulation of excitatory/inhibitory balance [68]. Therefore, more experimental evidence is needed to ask whether cholinergic neurons in the BF express *Oxtr*.

A recent study on the projectomes of oxytocin neurons in the paraventricular hypothalamic nucleus revealed abundant oxytocinergic innervation in some subregions within the BF like LS, MS, and diagonal band nucleus [69]. The potential synaptic connections between these oxytocin neurons and *Oxtr*^+^ BF neurons within these regions warrant further investigation. In addition, both cholinergic neurons and GABAergic neurons in the BF can project to various cortical and subcortical regions [30], releasing acetylcholine or GABA, neurotransmitters crucial for neural development [70, 71]. Notably, we found that *Oxtr* expression peaks at P14 in the BF, a critical period for synapse development and the excitatory-to-inhibitory switch in GABA effects [44, 56]. Therefore, it is imperative to identify the specific circuits expressing *Oxtr* in the BF and investigate whether *Oxtr* influences the development of downstream brain areas.

The *Oxtr* expression pattern in the hippocampus has a significant dorsal-ventral difference, with the ventral hippocampus expressing more *Oxtr* than the dorsal part. In addition, notable differences are also present in the distribution of *Oxtr* among cell types, especially in the DG, with higher levels of *Oxtr* in glutamatergic neurons in the vDG than in the dDG. This pattern may correlate with its functional heterogeneity. The dorsal hippocampus is predominantly involved in learning and memory, whereas the ventral region is more associated with motivational and emotional behaviors [72]. The ventral hippocampus has been acknowledged for its extensive involvement in the regulation of anxiety [73]. Given that oxytocin is a well-established anxiety regulator, the ventral hippocampus could be its important target. In addition, a recent study has revealed that OXTR in the mossy cells in the vDG is crucial to social discrimination [74]. This finding suggests a potential key role for *Oxtr* in social motivation and emotion processing. Moreover, the distinct connectivity patterns of the dorsal and ventral hippocampus are noteworthy. A typical example is that the ventral rather than the dorsal hippocampus has extensive reciprocal connections with the olfactory bulb, piriform cortex, and amygdala [72, 75], aligning with the expression pattern of *Oxtr*. Consequently, while the ventral hippocampus is directly implicated in olfactory threat memory, the dorsal hippocampus is not similarly engaged [75]. Correspondingly, oxytocin has been extensively reported to be involved in fear regulation [76]. This finding also suggests the importance of OXTR in olfactory processing. In addition, serotonin receptors are differentially distributed along the dorsal-ventral axis of the hippocampus [77], which may suggest an interplay between the oxytocin system and the serotonin system. Although various functions of *Oxtr* in the hippocampus have been reported [11, 12, 78], most studies have either focused exclusively on the dorsal part or have not differentiated between the dorsal and ventral regions. Our study underscores the necessity of distinguishing between the dorsal and ventral hippocampus and highlights the importance of investigating the ventral region. Furthermore, we noted that *Oxtr* expression in the ventral CA2/CA3 and adjacent areas, such as the entorhinal cortex and the amygdalohippocampal area, peaks at P28. Social interaction behavior is shaped developmentally, with both sociability and social novelty preference increasing during adolescence [79]. Interventions during adolescence can significantly impair social novelty preference in adulthood [79]. Given the importance of puberty in these processes [80], *Oxtr* in the ventral hippocampus may play a role in the development of social behavior.

We found a development-dependent effect of sex on the expression of *Oxtr* in the amygdaloid complex, with larger variances presenting at P14. This phenomenon may be associated with the sexually different developmental patterns of expression of OXTR. Recent research on human *OXTR* has shown that its expression in males peaks in the early postnatal stage, while in females, it shows a major peak around birth and a lower peak in the later postnatal period [81]. The later postnatal peak in females may cause higher *Oxtr* levels in males at P14. However, the sexual dimorphism in the medial amygdala which has been reported [48, 82] was not found at P28 and P56. One possible reason may be the different location; the medial amygdala is a larger region with several subregions and only the anterior ventral part was analyzed in our study, so different subregions might have distinct expression patterns. In addition, some previous studies have also reported other sexually dimorphic regions. For instance, the piriform cortex and the anteroventral periventricular nucleus have higher OXTR levels in female mice [15, 17], while the ventral premammillary nucleus has a higher OXTR level in male mice [17]. Therefore, more comprehensive data from females are essential to enhance our understanding of the oxytocin system.

A previous study provided a detailed description of OXTR expression patterns using reporter mice [17]. Consistent with our results, this study found that OXTR levels peak during early postnatal periods and subsequently decrease. However, some discrepancies were noted, including different cortical peak time points. These variations may arise from distinct methodological approaches. We applied RNAscope ISH to directly visualize *Oxtr* mRNA and no post-transcriptional processes were involved. However, previous reports have indicated that post-transcriptional processes can impact OXTR levels. For instance, the hippocampus and cerebellum exhibit almost identical OXTR protein levels, despite the cerebellum having lower *Oxtr* mRNA levels than the hippocampus [83]. Thus, our results align with another study using RNA sequencing [15]. In addition, our study delineates *Oxtr* expression patterns from a distinct perspective, focusing on the abundance of *Oxtr* mRNA, whereas the previous study concentrated on the density of *Oxtr*^+^ cells [17].

The study applying immunofluorescence detected clear signals in CA1 [15], while our results indicate minimal expression of *Oxtr* in this area. Apart from the impact of post-transcriptional processes, the subcellular localization may also influence the detection of *Oxtr*. *Oxtr* has been reported to be localized both presynaptically and postsynaptically [15], which suggests that the *Oxtr* signal detected by immunofluorescence in CA1 could originate from projection neurons in upstream regions. The mismatches in the localization of *Oxtr* mRNA and OXTR protein have also been reported in the prairie vole brain. Specifically, while *Oxtr* mRNA is confined to the SP, OXTR protein labeling is evident both superficially and basally to the SP in the hippocampus, which may be due to the dendritic localization of OXTR. Moreover, the substantia nigra pars reticulata shows robust protein labeling without corresponding mRNA signals, potentially due to the presynaptic localization of OXTR in neurons projecting from the upstream nucleus accumbens [19].

In addition, a recent study provided a high-resolution transcriptomic and spatial atlas of cell types in the whole mouse brain and a platform for single-cell data in the Allen Brain Cell Atlas (https://knowledge.brain-map.org/). Interestingly, some of our results can be validated in the database from the Allen Brain, such as the higher expression of *Oxtr* in cortical GABAergic neurons, cortical glutamatergic neurons in L2/3, and the amygdaloid complex. It should be noted that although *Oxtr* is mainly expressed in neurons based on the Allen Brain database, it is expressed in glia at a relatively lower level in the adult mouse brain (https://knowledge.brain-map.org/). Therefore, additional experimental evidence on the expression and function of *Oxtr* in glia is warranted in future work. Moreover, it is well-established that GABAergic neurons are highly heterogeneous with various electrophysiological properties and behavioral effects [27]. Previous studies have uncovered OXTR expression in SST^+^ cells in the prefrontal cortex [24], somatosensory cortex [10], and hippocampus [11]. However, in this study, GABAergic neurons were not classified into subtypes. Further research is expected to compare the variance in OXTR expression among different GABAergic neurons.

Another main limitation of our study is the incomplete coverage of brain regions. Firstly, only 9-12 slices per mouse were selected for RNAscope ISH, which resulted in the omission of certain brain regions including established OXTR-expressing areas such as the nucleus tractus solitarius and the ventral tegmental area. In addition, the limited number of sections means that some regions were not represented at their maximal cross-sectional area. Secondly, brain regions were identified manually in our study. Although this manual approach aims to mitigate structural variances among brains from different developmental stages, it may cause inaccurate boundaries and restrict the number of identifiable regions. In some regions such as the medulla, due to the lack of clear boundaries, none of the regions were identified.

It is notable that the high sensitivity of RNAscope ISH has two sides, especially in the statistic of positive cell ratios. The correlation between the number of *Oxtr* mRNA puncta within a cell and the classification of that cell as *Oxtr*^+^ can sometimes be ambiguous. A more refined approach would be to categorize and analyze cells based on the number of puncta they contain. However, such complex statistical methods are not feasible for large-scale analysis. Consequently, in this study, the ratio of positive cells was obtained automatically by the software based on a cut-off classifying any cell with *Oxtr* mRNA puncta as *Oxtr*^+^, which means some positive cells might fall below the threshold used by other methods. Although this approach may yield results that vary from those obtained by other techniques, our study offers a novel perspective on the distribution of *Oxtr*.

## Conclusion

In conclusion, this study presents a detailed spatiotemporal mapping of *Oxtr* expression in the postnatal brains of male mice, providing high-resolution insights. We meticulously delineate the patterns of *Oxtr* expression in various key regions, including the cortex, BF, hippocampus, and amygdaloid complex. Notably, within the cortical layers, the GABAergic neurons in L5 are one of the neuronal populations with the highest *Oxtr* levels in any cortex at any developmental stage, and we also found a depth-dependent variance of *Oxtr* expression patterns in L2/3. In the BF, *Oxtr* expression is particularly pronounced in *Vgat *^+^ cells. The hippocampus demonstrates a clear dorsal-ventral differential pattern in *Oxtr* expression. In addition, within the amygdaloid complex, we report subregion-specific patterns of *Oxtr* expression. Collectively, these findings provide a novel perspective on the distribution of *Oxtr* and lay the groundwork for future research into the functional roles of *Oxtr* in neural processes.

## Supplementary Information

Below is the link to the electronic supplementary material.

Supplementary file 1 (PDF 4007 KB)

Supplementary file 2 (XLSX 29 KB)

## Data Availability

All relevant data of this study are available from the corresponding authors upon reasonable request.
