# Supplementary material for: Spatiotemporal Mapping of the Oxytocin Receptor at Single-Cell Resolution in the Postnatally Developing Mouse Brain
Source: Neurosci Bull. 2024 Sep 15;41(2):224–42. doi: 10.1007/s12264-024-01296-x (PMC11794781; doi:10.1007/s12264-024-01296-x)
Supplement: Supplementary file 1 — (PDF 4007 KB) [file 12264_2024_1296_MOESM1_ESM.pdf]

Supplementary Information

Supplementary Figures

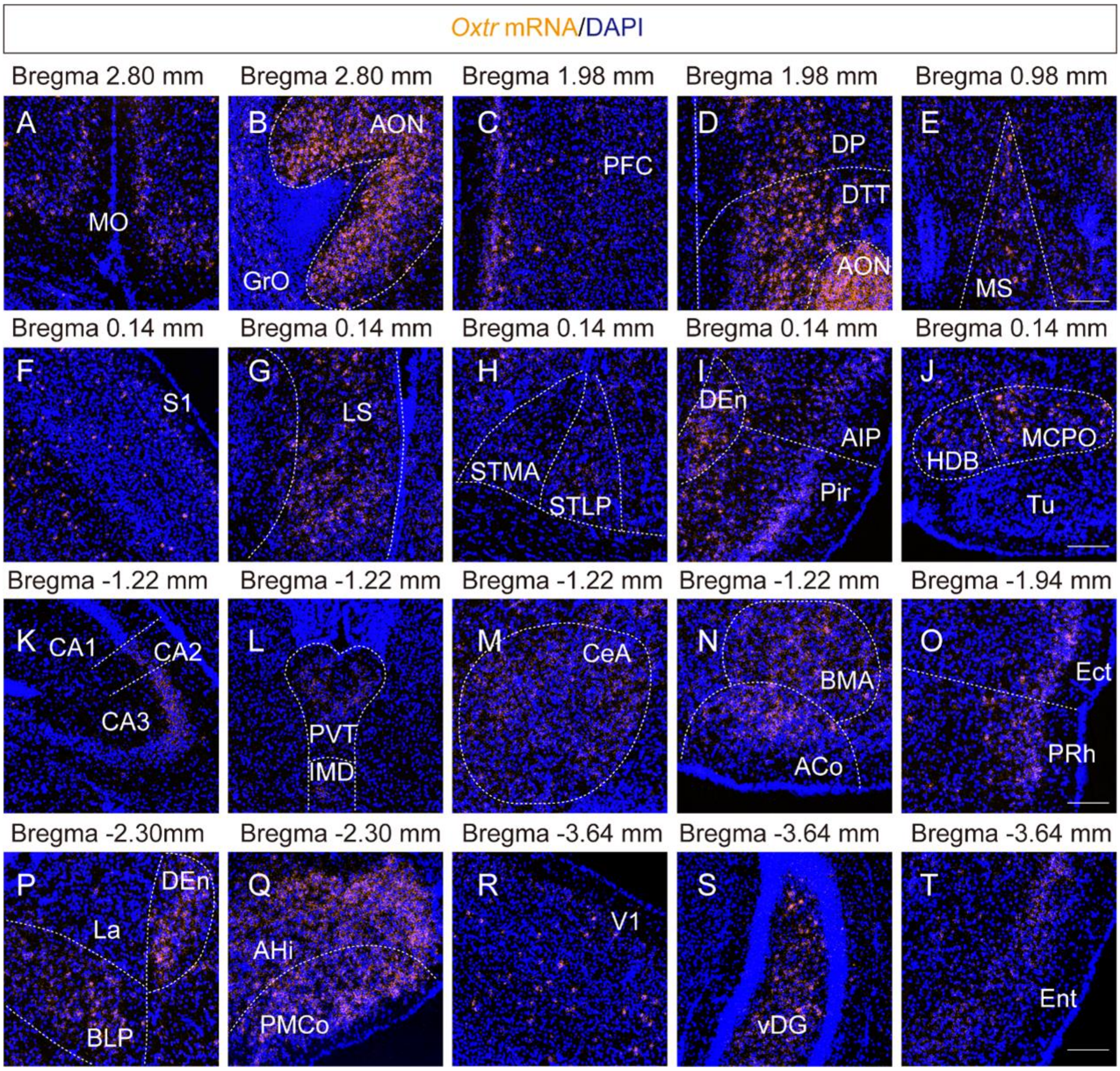

Fig. S1 The expression pattern of *Oxtr* in the male mouse brain at P14. **A-T** Representative images of the medial orbital cortex **(A)**, olfactory bulb **(B)**, prefrontal cortex **(C)**, dorsal peduncular cortex and dorsal taenia tecta **(D)**, medial septal nucleus **(E)**, primary somatosensory cortex **(F)**, lateral septal nucleus **(G)**, bed nucleus of the stria terminalis **(H)**, insular cortex and piriform cortex **(I)**, nucleus of the diagonal band and magnocellular preoptic nucleus **(J)**, hippocampus **(K)**, paraventricular thalamic nucleus **(L)**, central amygdaloid nucleus **(M)**, basomedial amygdaloid nucleus and anterior cortical amygdaloid area **(N)**, ectorhinal cortex and perirhinal cortex **(O)**, dorsal endopiriform nucleus and basolateral amygdaloid nucleus, posterior part **(P)**, amygdalo-hippocampal area and posteromedial cortical amygdaloid area **(Q)**, primary visual cortex **(R)**, ventral DG **(S),** and entorhinal cortex **(T)**. The full names of all abbreviations are listed in Table S1.

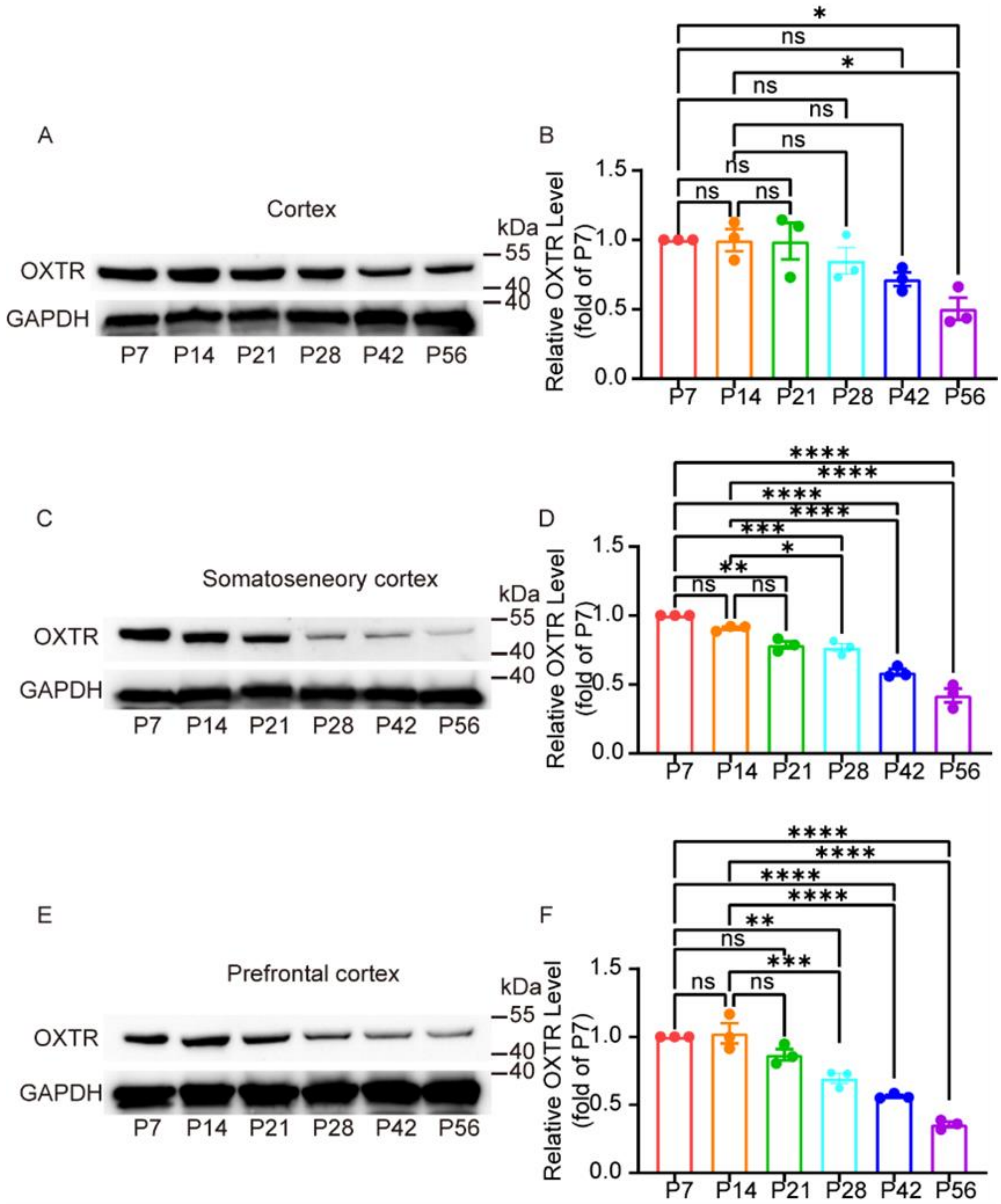

Fig. S2 The expression trajectories of OXTR protein in the cortex. **A-F** Representative Western blots and autoradiograms with quantitative results for the expression trajectories of OXTR in the entire cortex **(A, B)**, somatosensory cortex **(C, D)** and prefrontal cortex **(E, F)** (*n* = 3). Data are presented as the mean ± SEM.

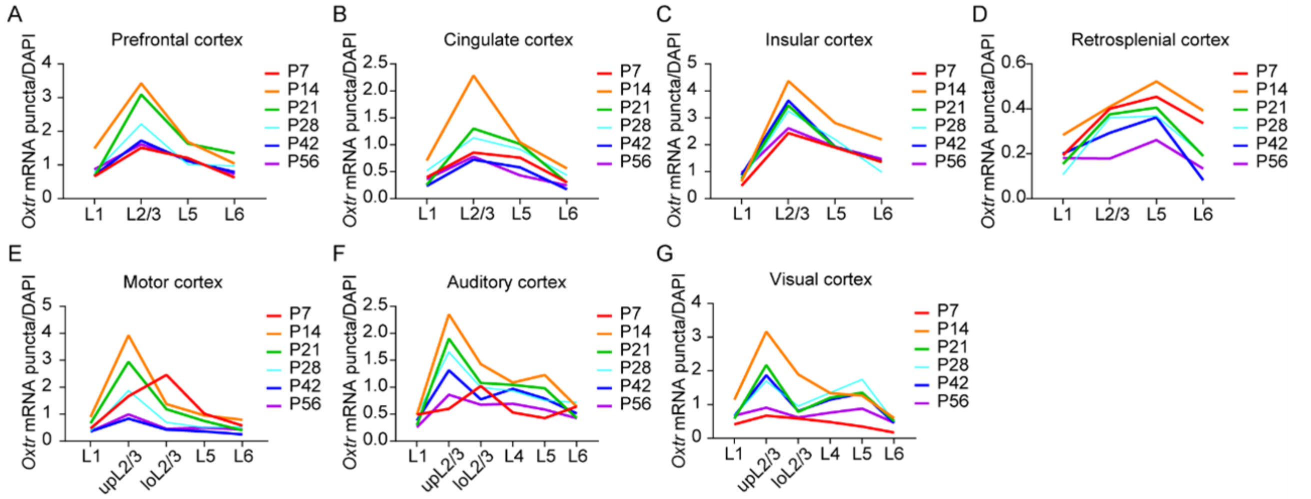

Fig. S3 The *Oxtr* expression trajectories of each layer in different cortices. **A-G** The *Oxtr* expression trajectories of different layers in the prefrontal **(A)**, cingulate **(B)**, insular **(C)**, retrosplenial **(D)**, motor **(E)**, auditory **(F)**, and visual cortices **(G)** (n = 5). Data are presented as the mean.

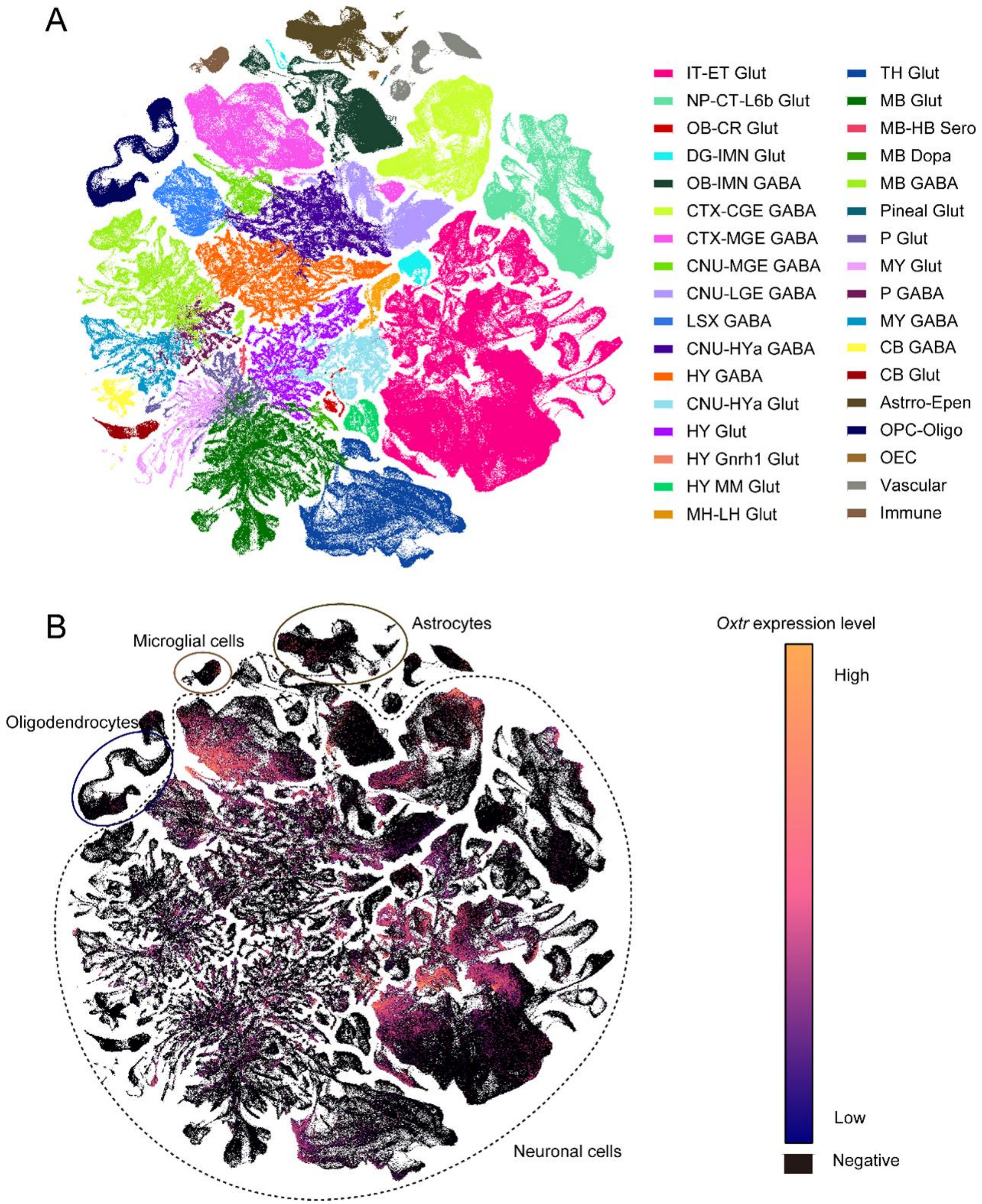

Fig. S4 The expression patterns of Oxtr in different cell types. **A** UMAP plot of single-cell data colored by different cell classes. **B** UMAP plot of single-cell data colored by the expression level of Oxtr. (the figures are downloaded and modified from https://knowledge.brain-map.org/)

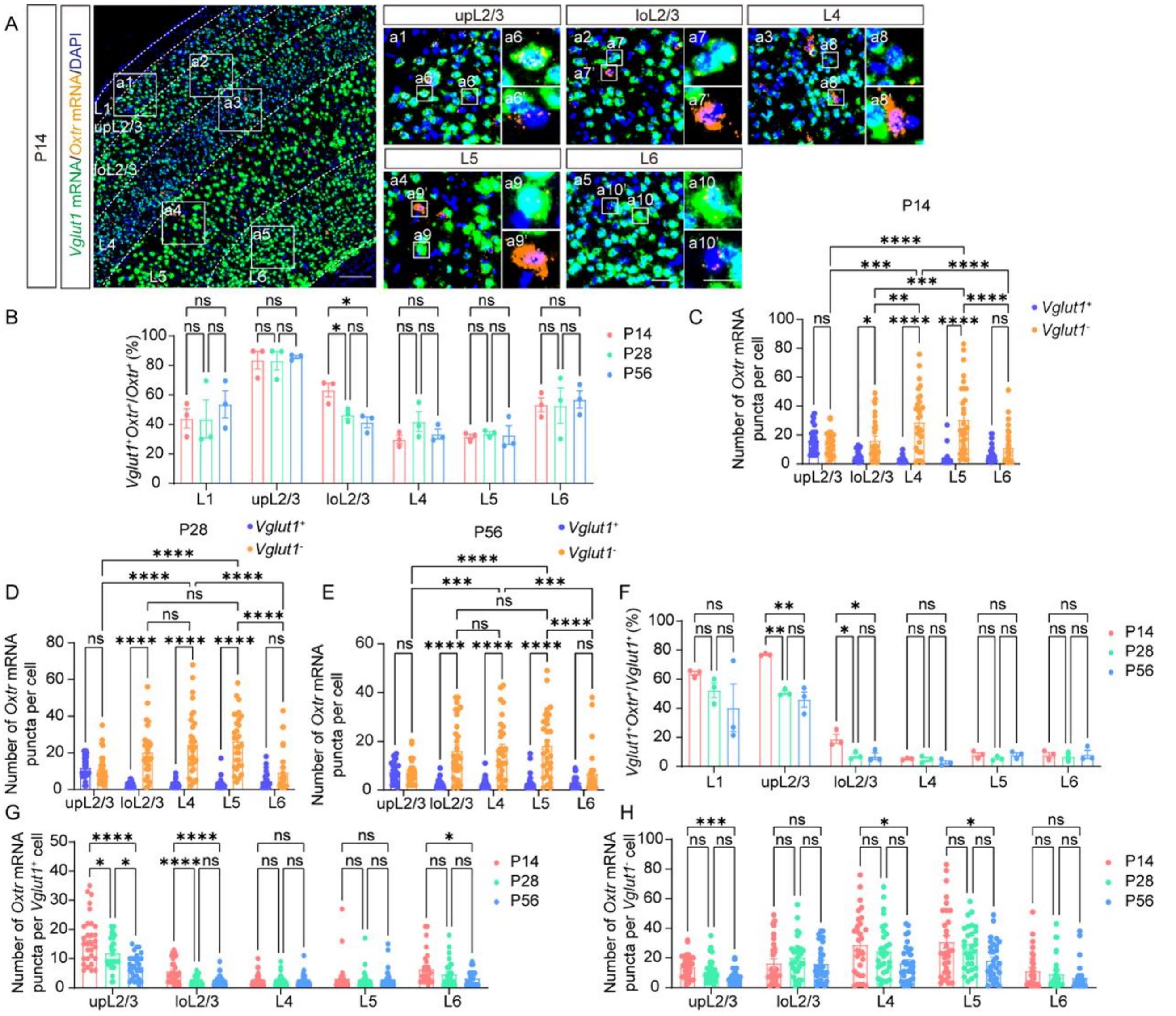

Fig. S5 The expression pattern of *Oxtr* in *Vglut1*$^{+}$ cells and *Vglut1*$^{-}$ cells in the somatosensory cortex. **A** Representative images of *Oxtr* mRNA (orange) co-labeling with *Vglut1* mRNA (green) from the somatosensory cortex at P14. Scale bar, 200 µm. **a1-a5** Representative images of each layer. Scale bar, 50 µm. **a6-a10** Representative images of *Vglut1*$^{+}$ cells. **a6'-a10'** Representative images of *Vglut1*$^{-}$ cells. Scale bar, 20 µm. **B** The ratio of *Vglut1*$^{+}$ cells among *Oxtr*$^{+}$ cells of each layer (n = 3). **C-E** The number of *Oxtr* mRNA puncta in different cell types and layers from somatosensory cortex at P14 **(C)**, P28 **(D)**, and P56 **(E)** (n = 30 cells, 3 mice). **J** The ratio of *Oxtr*$^{+}$ cells among *Vglut1*$^{+}$ cells of each layer (n = 3). **G**, **H** Developmental comparison of the number of *Oxtr* mRNA puncta in *Vglut1*$^{+}$ cells **(G)** and *Vglut1*$^{-}$ cells **(H)** of each layer (n = 30 cells, 3 mice). Data are presented as the mean ± SEM. **P* <0.05, ***P* <0.01, ****P* <0.001, *****P* <0.0001, one-way ANOVA with Tukey's multiple comparisons test **(B, F-H)** or two-way ANOVA with Tukey's multiple comparisons test **(C-E)**.

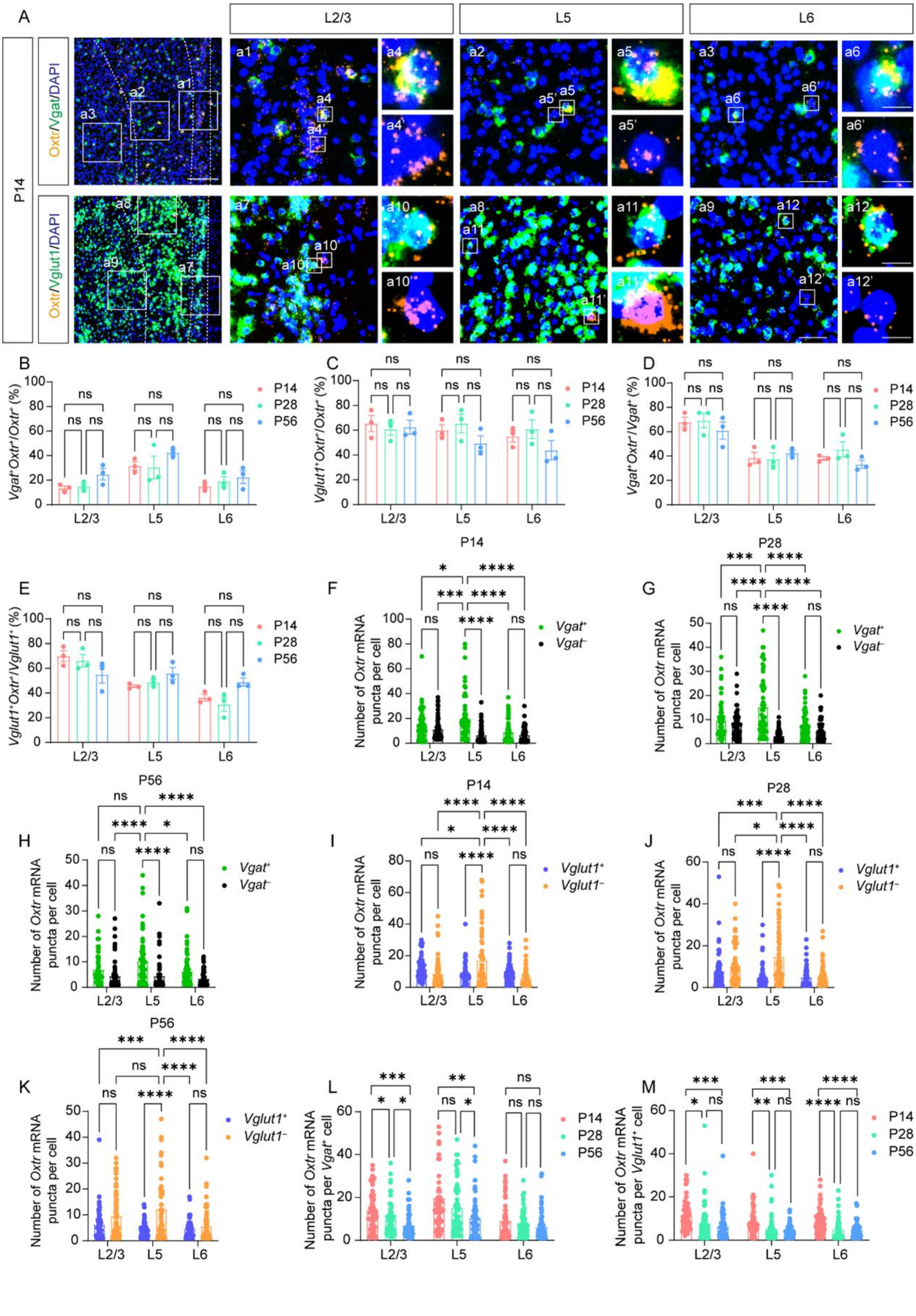
A
P14
L2/3
L5
L6
Oxtr/Vgat/DAPI
Oxtr/Vglut1/DAPI
B
Vgat+Oxtr+/Oxtr+ (%)
C
Vglut1+Oxtr+/Oxtr+ (%)
D
Vgat+Oxtr+/Vgat+ (%)
E
Vglut1+Oxtr+/Vglut1+ (%)
P14
P28
P56
F
G
H
Number of Oxtr mRNA puncta per cell
Vgat+
Vgat−
I
J
K
Vglut1+
Vglut1−
L
Number of Oxtr mRNA puncta per Vgat+ cell
M
Number of Oxtr mRNA puncta per Vglut1+ cell
ns

Fig. S6 The expression pattern of *Oxtr* in the prefrontal cortex. **A** Representative images of *Oxtr* mRNA (orange) co-labeling with *Vgat* mRNA or *Vglut1* mRNA (green) at P14. Scale bar, 200 μm. **a1-a3, a7-a9** Representative images of each layer. Scale bar, 50 μm. **a4-a6** Representative images of $Vgat^+$ cells. **a4'-a6'** Representative images of $Vgat^-$ cells. **a10-a12** Representative images of $Vglut1^+$ cells. **a10'-a12'** Representative images of $Vglut1^-$ cells. Scale bar, 20 μm. **B** The ratio of $Vgat^+$ cells among $Oxtr^+$ cells in each layer (n = 3). **C** The ratio of $Vglut1^+$ cells among $Oxtr^+$ cells in each layer (n = 3). **D** The ratio of $Oxtr^+$ cells among $Vgat^+$ cells in each layer (n = 3). **E** The ratio of $Oxtr^+$ cells among $Vglut1^+$ cells in each layer (n = 3). **F-K** The number of *Oxtr* mRNA puncta in different cell types and layers from somatosensory cortex at P14 **(F, I)**, P28 **(G, J)**, and P56 **(H, K)** (n = 60 cells, 3 mice). **L, M** Developmental comparison of the number of *Oxtr* mRNA puncta in $Vgat^+$ cells **(L)** and $Vglut1^+$ cells **(M)** in each layer (n = 60 cells, 3 mice). Data are presented as the mean ± SEM. **P* <0.05, ***P* <0.01, ****P* <0.001, *****P* <0.0001, one-way ANOVA with Tukey's multiple comparisons test **(B-E, L, M)** or two-way ANOVA with Tukey's multiple comparisons test **(F-K)**.

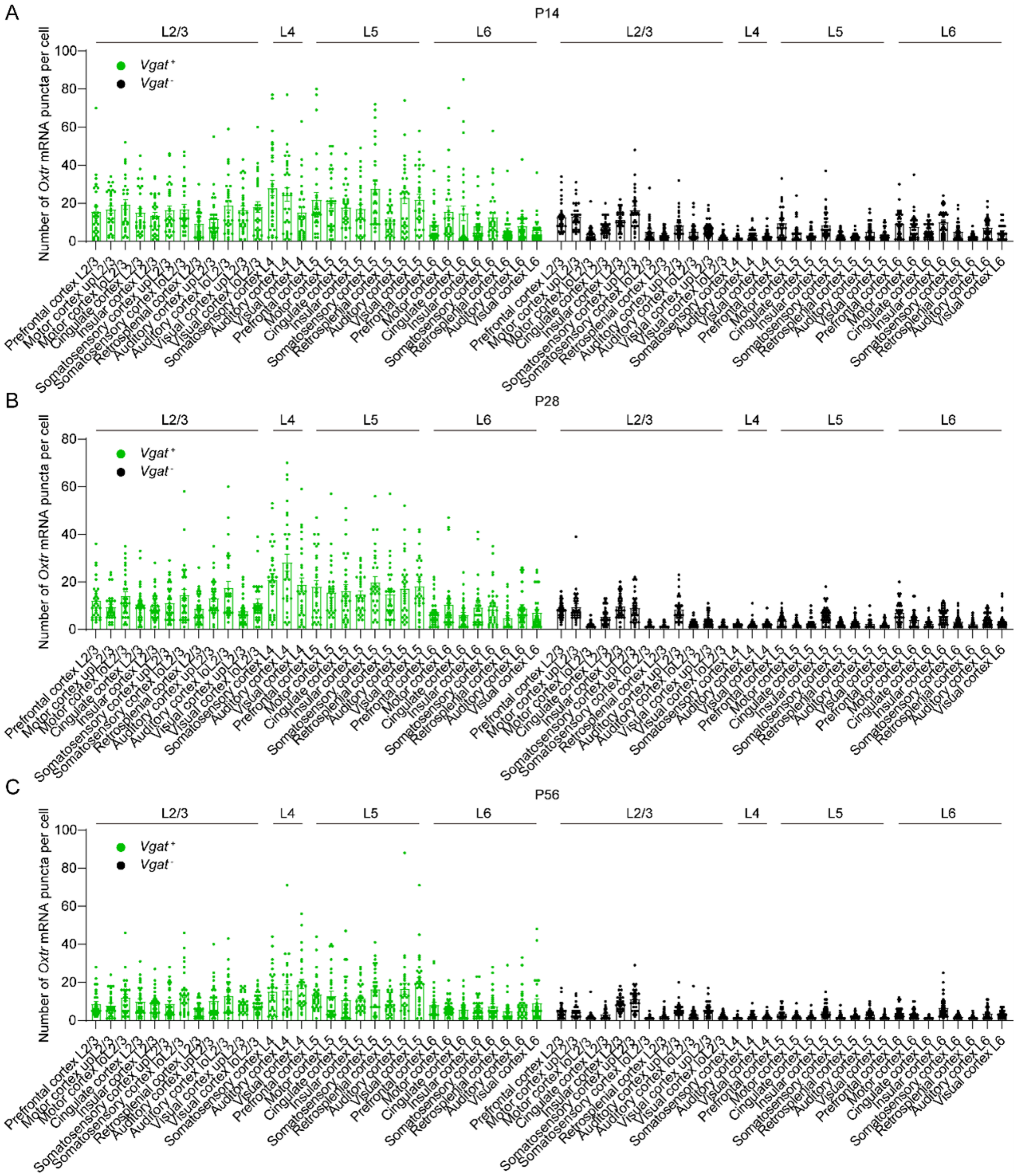

Fig. S7 Layered *Oxtr* distributions between *Vgat*$^{+}$ cells and *Vgat*$^{-}$ cells in different cortices. **A-C** The number of *Oxtr* mRNA puncta in different cell types and layers from different cortices at P14 **(A)**, P28 **(B)**, and P56 **(C)**. Data are presented as the mean ± SEM.

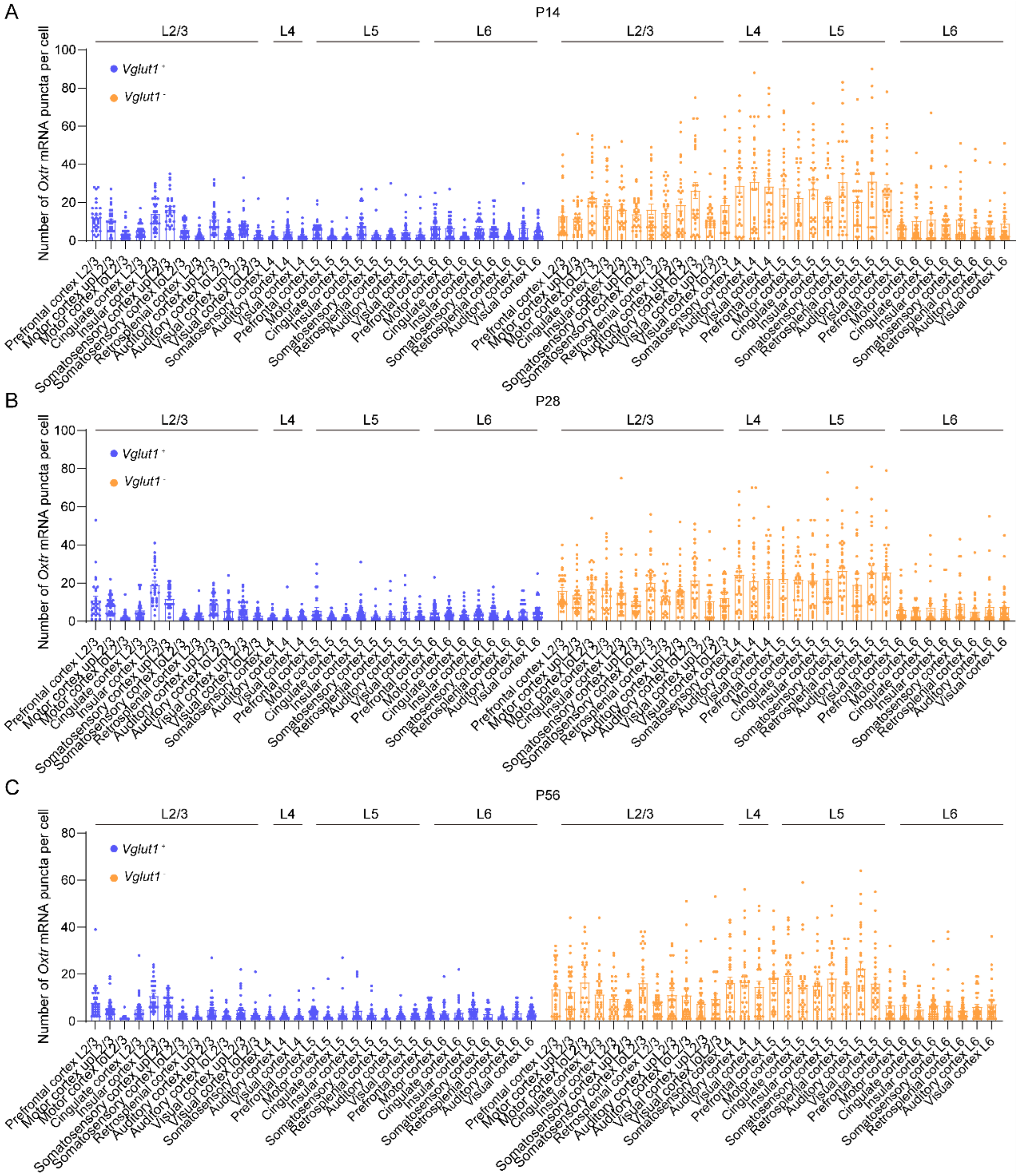

Fig. S8 Layered *Oxtr* distributions between *Vglut1*$^{+}$ cells and *Vglut1*$^{-}$ cells in different cortices

A-C The number of *Oxtr* mRNA puncta in different cell types and layers from different cortices at P14 **(A)**, P28 **(B)**, and P56 **(C)**. Data are presented as the mean ± SEM.

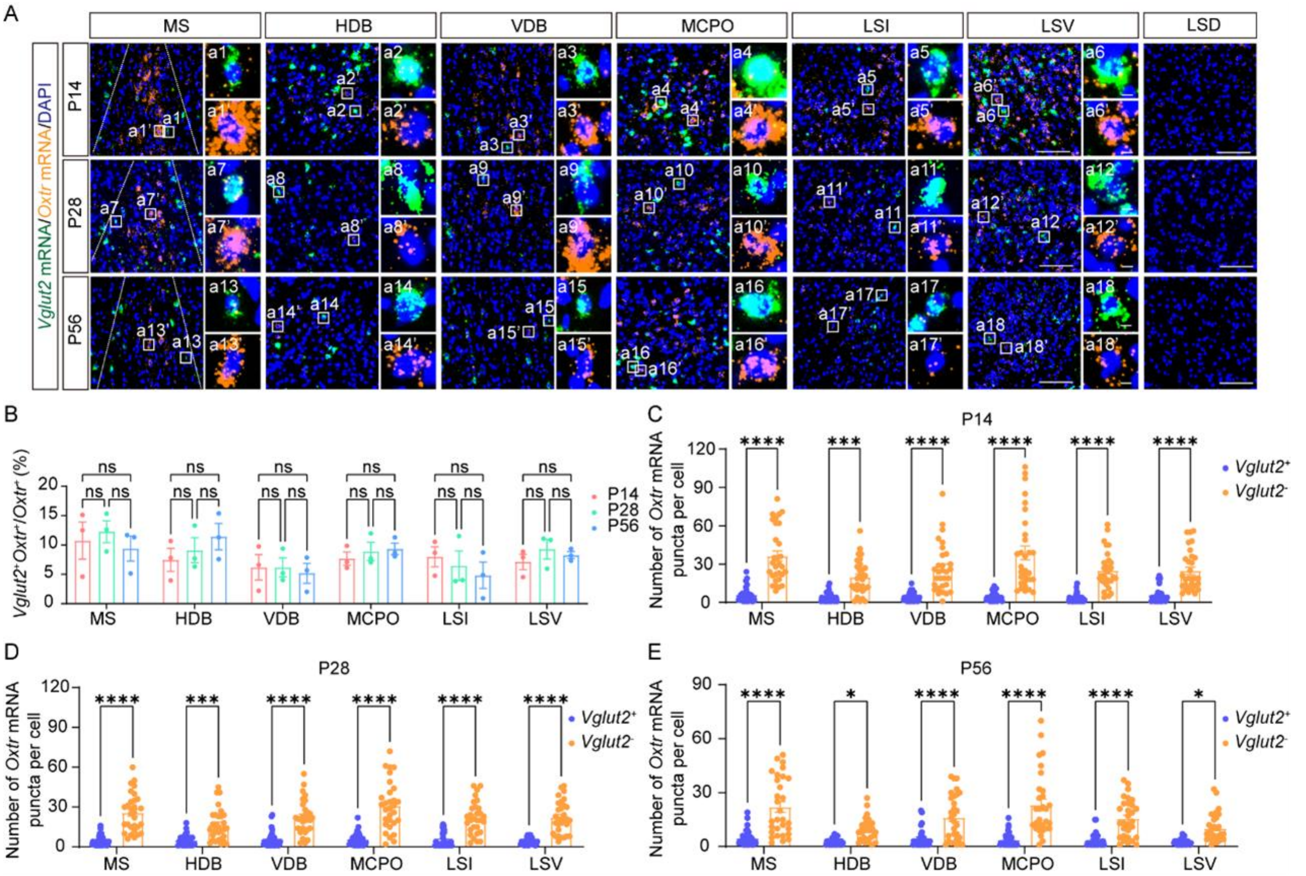

Fig. S9 The expression pattern of *Oxtr* in *Vglut2*$^+$ cells and *Vglut2*$^–$ cells in the basal forebrain. **A** Representative images of *Oxtr* mRNA (orange) co-labeling with *Vglut2* mRNA (green) from the BF at different periods (No *Vglut2* signal was detected in the dorsal part of the lateral septal nucleus, thus is not included). Scale bar, 100 µm. **a1-a18** Representative images of *Vglut2*$^+$ cells. Scale bar, 5 µm. **a1'-a18'** Representative images of *Vglut2*$^–$ cells. Scale bar, 5 µm. **B** The ratio of *Vglut2*$^+$ cells among *Oxtr*$^+$ cells of subregions in the BF (n = 3). **C-E** The number of *Oxtr* mRNA puncta in different cell types and subregions from the BF at P14 **(C)**, P28 **(D)**, and P56 **(E)** (n =30 cells, 3 mice). Data are presented as the means ± SEM. *P <0.05, ****P <0.0001, one-way ANOVA with Tukey's multiple comparisons test **(B)** or unpaired t-test **(C-E)**. The full names of all abbreviations are listed in Table S1.

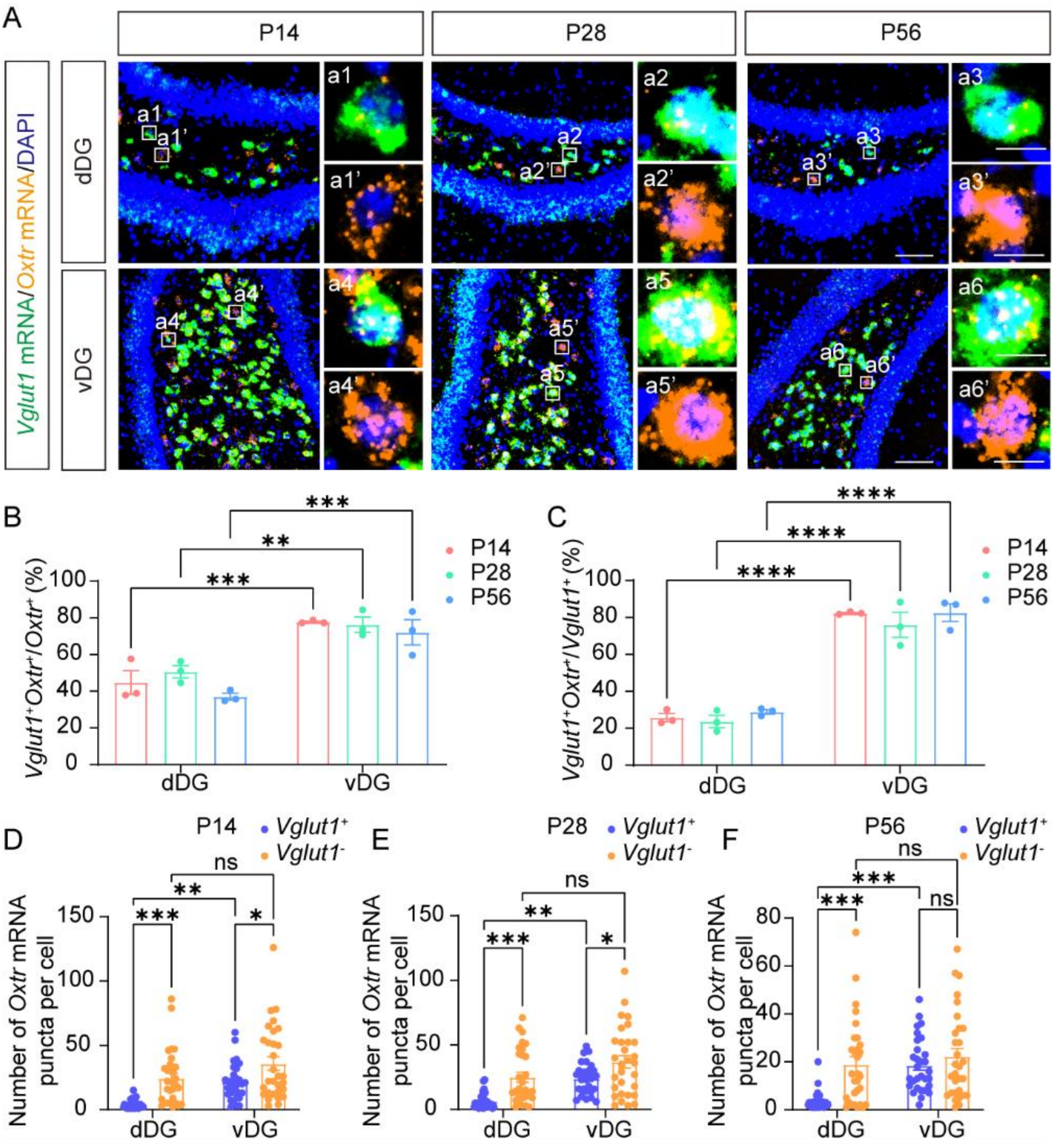

Fig. S10 The expression patterns of *Oxtr* in *Vglut1*$^{+}$ cells and *Vglut1*$^{-}$ cells in the dorsal and ventral DG. **A** Representative images of *Oxtr* mRNA (orange) co-labeling with *Vglut1* mRNA (green) at different developmental stages. Scale bar, 100 μm. **a1-a6** Representative images of *Vglut1*$^{+}$ cells. **a1'-a6'** Representative images of *Vglut1*$^{-}$ cells. Scale bar, 20 μm. **B** The ratio of *Vglut1*$^{+}$ cells among *Oxtr*$^{+}$ cells (n =3). **C** The ratio of *Oxtr*$^{+}$ cells among *Vglut1*$^{+}$ cells (n = 3). **D-F** The number of *Oxtr* mRNA puncta in different cell types and regions from the DG at P14 **(D)**, P28 **(E)**, and P56 **(F)** (n = 30 cells, 3 mice). Data are presented as the mean ± SEM, **P <0.01, ****P <0.0001, two-way ANOVA with Tukey's multiple comparisons test **(D-F)** or unpaired t-test **(B, C).**

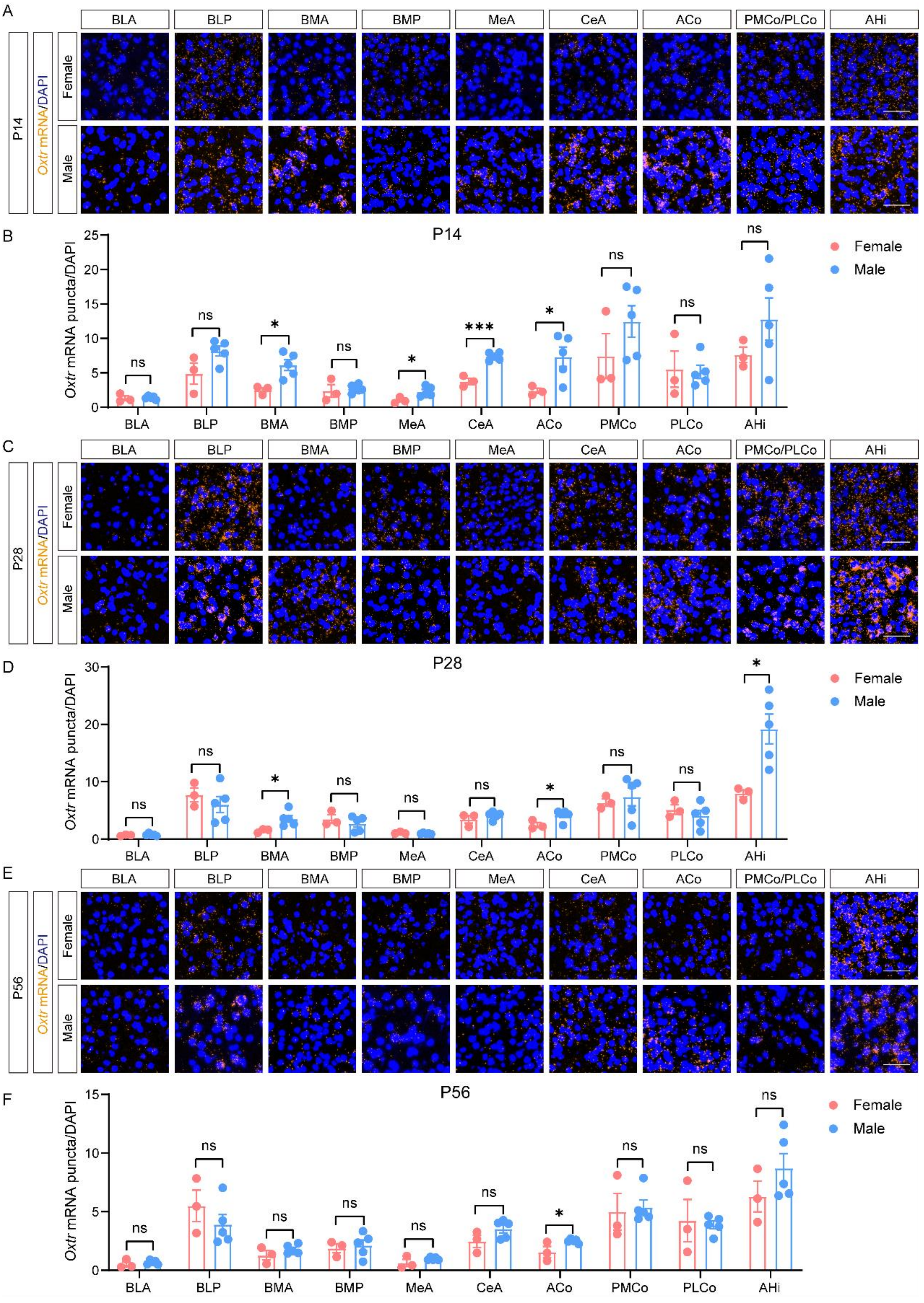

A
BLA
BLP
BMA
BMP
MeA
CeA
ACo
PMCo/PLCo
AHi
P14
Oxtr mRNA/DAPI
Female
Male
B
P14
Oxtr mRNA puncta/DAPI
Female
Male
ns
*
***
C
P28
D
P28
E
P56
F
P56
PMCo
PLCo

Fig. S11 The effect of sex on the *Oxtr* expression pattern in the amygdaloid complex. **A-F** Representative images and quantitative analyses of *Oxtr* expression levels between females and males at P14 **(A, B)**, P28 **(C, D)** and P56 **(E, F)** (n = 3 for females, n = 5 for males). Scale bar, 50 μm. Data are presented as the mean ± SEM, *P <0.05, ***P <0.001, unpaired t-test **(B, D, F).**
